# QuimP: analyzing transmembrane signalling in highly deformable cells

**DOI:** 10.1093/bioinformatics/bty169

**Published:** 2018-03-16

**Authors:** Piotr Baniukiewicz, Sharon Collier, Till Bretschneider

**Affiliations:** Department of Computer Science & Zeeman Institute, University of Warwick, Coventry, UK

## Abstract

**Summary:**

Transmembrane signalling plays important physiological roles, with G protein-coupled cell surface receptors being particularly important therapeutic targets. Fluorescent proteins are widely used to study signalling, but analyses of image time series can be challenging, in particular when cells change shape. QuimP software semi-automatically tracks spatio-temporal patterns of fluorescence at the cell membrane at high spatial resolution. This makes it a unique tool for studying transmembrane signalling, particularly during cell migration in immune or cancer cells for example.

**Availability and implementation:**

QuimP (http://warwick.ac.uk/quimp) is a set of Java plugins for Fiji/ImageJ (http://fiji.sc) installable through the Fiji Updater (http://warwick.ac.uk/quimp/wiki-pages/installation). It is compatible with Mac, Windows and Unix operating systems, requiring version >1.45 of ImageJ and Java 8. QuimP is released as open source (https://github.com/CellDynamics/QuimP) under an academic licence.

**Supplementary information:**

Supplementary data are available at *Bioinformatics* online.

## 1 Introduction

In transmembrane signalling the cell membrane plays a fundamental role in localizing intracellular signalling components to specific sites of action, for example to reorganize the actomyosin cortex during cell polarization and locomotion. The localization of different components can be directly or indirectly visualized using fluorescence microscopy, for high-throughput screening commonly in 2D. A quantitative understanding demands segmentation and tracking of cells and fluorescence signals at the membrane, for example those associated with actin polymerization at the cell front of locomoting cells. Different tools for cell segmentation and tracking are reviewed in (Barry *et al.*, 2015; Meijering *et al.*, 2009; Ryan *et al.*, 2013). Segmentation can employ thresholding, region growing, active contours or level sets, to obtain closed cell contours and sample fluorescence adjacent to the cell edge in a straightforward manner. The most critical step however is cell edge tracking, which links points on contours at time *t* to corresponding points at *t *+* *1. Optical flow methods exist, but usually fail to meet the requirement that total fluorescence must not change. QuimP uses a unique method [ECMM, electrostatic contour migration method (Tyson *et al.*, 2010)] which has been shown to outperform traditional level set methods, and works at subpixel resolution. ECMM minimizes the sum of path lengths connecting all pairs of points, equivalent to minimizing the energy required for cell deformation. QuimP’s active contour based segmentation and outline tracking algorithms have been described in Dormann *et al.* (2002) and Tyson *et al.* (2010, 2014). It has been developed on and off since 2002, but recent funding resulted in a completely reengineered and redesigned, sustainable software that is accessible to a much wider circle of non-expert users.

## 2 New features

**Fig. 1.**
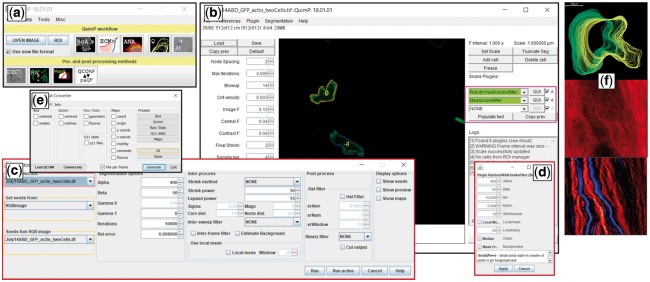
QuimP GUI for analyzing cell motility. (**a**) QuimP toolbar, with tools arranged in the order of a typical workflow. Upper row: Open image time series, and main data analysis plugins (BOA: cell segmentation, ECMM: contour tracking, ANA: sampling of cortical fluorescence, QA: detailed quantitative analysis and visualization in the form of spatial-temporal maps, PA: protrusion analysis (experimental, working Matlab routines are provided)). Bottom row: Pre- and post-processing plugins (DIC: DIC image reconstruction, RW: customized random walk segmentation, Mask: Cell outline to mask converter). (**b**) BOA segmentation window with novel feature of external contour filters. (**c**) Interface for the new random walk segmentation module. (**d**) New BOA plugin that integrates random walk and active contour segmentation. (**e**) Conversion tool to export csv files. (**f**) Exemplary results from the QA module: cell outlines, fluorescence map, convexity map

QuimP has recently been released as open source (current version 2018-02-01, user manual available on homepage; new features including walkthrough examples are detailed in Supplementary Material SI-A). Figure 1 shows the graphical user interface. Main new features are: (i) use of the JSON format to store complete analysis workflows in QCONF (QuimP Configuration) files, and facilitate exchange of data with other programming languages (SI-B); also particularly useful when segmenting long sequences that require manual corrections. (ii) Four new modules have been added, for reconstructing differential interference contrast (DIC) images, customized random walk cell segmentation (with advanced tracking), generation of image masks from segmented cell outlines, and exporting data in comma-separated value format, for example for importing data to other phenotypic cell analysis pipelines. (iii) A new architecture supports custom vector filters that directly operate on cell contour data, without requiring deep knowledge of QuimP; Examples are a running mean filter, or a protrusion removal filter (SI-A). (iv) Segmentation masks generated by other ImageJ methods or external software can be used as input for further QuimP analysis (SI-A). (v) Improved segmentation by combining QuimP’s original active contour (AC) segmentation with a modified random walk (RW) method (Fig. 1b): Active contour methods are good at segmenting cells, but notoriously struggle when dealing with highly concave cell outlines. RW (Grady, 2006) is superior in this respect, but has problems with strong gradients in fluorescence, as typically observed for many proteins involved in cell polarization and directed cell movement. QuimP includes a locally adaptive version which overcomes this problem and compares favourably to other top-rated tools (SI-C).

RW is a supervised learning method that requires users to label (seed) a small number of foreground (cell) and background image pixels. QuimP now employs preliminary AC segmentation masks to seed class labels for each frame of a time series automatically (SI-A and SI-C). Foreground and background pixels are assigned after contracting and expanding vectorized masks. Our algorithm preserves the shape of the contour and prevents that thin cellular processes are eroded (SI-D), significantly improving the segmentation of cellular protrusions and cavities.

Segmentation quality was evaluated using typical time-series of migrating cells tagged with fluorescent markers for different cytoskeletal proteins. 750 image frames were manually segmented (gold standard), and compared to results from: (i) QuimP run in an unsupervised manner, i.e. without changing parameters between frames, and (ii) the Trainable Weka ImageJ plugin (Arganda-Carreras *et al.*, 2017). Our new method significantly reduces the Hausdorff distance (maximum distance between the segmented contour and the gold standard) and importantly, the number of false positive pixels per contour length (SI-C and SI-D).

## 3 Conclusions and perspectives

The strength of QuimP’s contour tracking (ECMM) and its versatility and user-friendliness have resulted in >70 publications to date where it has been used (http://warwick.ac.uk/quimp/quimp-refs). Recent developments, namely making QuimP available as open source, with frequent updates and extended documentation, improvements in segmentation quality, and important changes to the architecture to support customized cell contour filters, make QuimP accessible to a much wider user base. Phenotypic analysis successfully employed multivariate cell descriptors for modelling transitions between discrete cellular states using hidden Markov models (Gordonov *et al.*, 2016; Held *et al.*, 2010). QuimP is complementary in such that it provides a detailed picture of cortical spatio-temporal dynamics, for example to fit continuous partial differential equation models of cell reorientation (Lockley *et al.*, 2015), or to predict the localization of cellular blebs (Collier *et al.*, 2017). QuimP’s high-quality segmentation and boundary tracking routines therefore can provide more complex and importantly dynamic features of subcellular (cortical) regions, which might be used to enhance the feature space in current other modelling frameworks for phenotypic analysis (Cooper *et al.*, 2015; Johnson *et al.*, 2015). Hermans *et al.* (2013) for example used morphodynamic profiling by QuimP in combination with cross-correlations, and Granger causality analyses performed in Matlab. Thus, they were able to discriminate between non-metastatic and metastatic breast cancer cells in terms of their motility efficiency and spatiotemporal synchronization. QuimP is routinely used to analyze time series of several hundreds of cells per experimental condition, for example to study changes in the motility of melanocytes during melanoma progression (Mescher *et al.*, 2017). Future development will integrate QuimP with Omero, an image database system (https://www.openmicroscopy.org/omero), to better support large scale screening assays.

## Funding

We thank BBSRC for funding QuimP development (BB/M01150X/1).

*Conflict of Interest*: none declared.

## Supplementary Material

Supplementary Data
