# Supplementary material for: QuimP: analyzing transmembrane signalling in highly deformable cells
Source: Bioinformatics. 2018 Mar 16;34(15):2695–7. doi: 10.1093/bioinformatics/bty169 (PMC6061833; doi:10.1093/bioinformatics/bty169)
Supplement: Supplementary Data [file bty169_supp.zip › bty169-suppl_data/Supplementary_material_A.docx]

Supplementary material A – New features and workflow examples

This supplementary material describes plugins available from the QuimP Toolbar, and new features available in the BOA module.

A detailed description of all features is contained in QuimP’s online manual available here: <http://warwick.ac.uk/quimpdoc>.

Test data and example files can be downloaded from <http://www.warwick.ac.uk/quimp/test_data/>.

Contents

[BOA module – new features 2](#_Toc506194287)

[Working with plugins - possible use case 2](#_Toc506194288)

[Restoring contours from masks 5](#_Toc506194289)

[Suggested workflow 5](#_Toc506194290)

[Additional modules 6](#_Toc506194291)

[DIC module 6](#_Toc506194292)

[Suggested workflow 7](#_Toc506194293)

[Mask Generator module 8](#_Toc506194294)

[Suggested workflow 8](#_Toc506194295)

[Merging cell contours and source image 9](#_Toc506194296)

[Random Walk Segmentation module 10](#_Toc506194297)

[Suggested workflow for manually labelled images 10](#_Toc506194298)

[Multiple cell segmentation 11](#_Toc506194299)

[Combined segmentation - utilize active contours for seed generation, and local mean feature 12](#_Toc506194300)

[Combined segmentation - use Random walk segmentation directly from BOA module 14](#_Toc506194301)

[References 16](#_Toc506194302)

# BOA module – new features

The BOA module, which performs cell segmentation of time series image data, usually is the entry point for each new QuimP analysis. BOA also produces the initial *QCONF* file that stores vectorised cell outlines and principal shape descriptors and features associated with them.

The process of segmentation is supposed to be fully automatic with minimal user interaction, but for low quality images it usually requires some manual corrections. Latest QuimP versions make it possible to save the current work state and restore it later. Thus, segmenting long time series, where more frames may need manual tuning is less tiring and error prone. Segmentation parameters can be set and stored in the file independently for every frame.

Another improvement introduced in this version are outline filters, available from the right panel of the BOA plugin. Filters operate directly on vectorised cell contours allowing for more sophisticated post-processing. Technically, those filters are separate files that are read when BOA is called for the first time, in the same way as external plugins are discovered and utilised by ImageJ. Therefore, the functionality of the BOA module can be easily extended by new specific methods for contour processing, without having to have a deeper understanding of BOA’s internal working. Filters are written in Java and utilise the QuimP Filter API, which is documented on the project’s site^[[1]](#footnote-2)^.

The latest version also fixes a number of bugs found in the BOA module.

## Working with plugins - possible use case

We provide an example segmentation of a short time series, *KZ5-240214-vAR1-dev5.5h-buffer-2-short.tif*^[[2]](#footnote-3)^. The configuration file *KZ5-240214-vAR1-dev5.5h-buffer-2-short.QCONF* can be opened in BOA using the following steps:

1. Open *KZ5-240214-vAR1-dev5.5h-buffer-2-short.tif* in Fiji.
2. Run the BOA module from Fiji’s *Plugins* menu.
3. Head to *File->Load global config* and select *KZ5-240214-vAR1-dev5.5h-buffer-2-short.QCONF* file. You may be warned that image opened with BOA does not match that one referenced in loaded configuration. This is due to storing absolute path names in configuration files to stay compatible with old versions of QuimP.
4. The BOA window will look similar to that shown in Fig. 1. The last edited frame, zoom status, and segmentation parameters are restored.


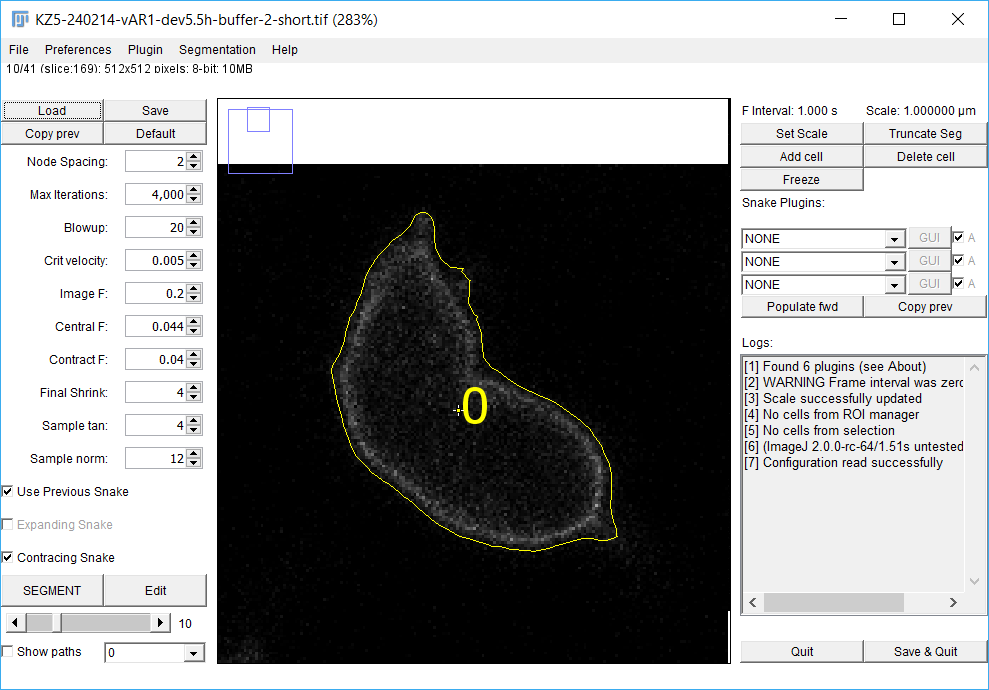


Fig. 1. Restored work state after loading KZ5-240214-vAR1-dev5.5h-buffer-2-short.QCONF configuration file.

1. We use the example to show how small-scale protrusions on the cell membrane can be filtered out using a novel HatSnakeFilter^[[3]](#footnote-4)^ which is available from the filter slots in the right panel. Such protrusions could be for example microspikes, filopodia, retraction fibres, or blebs. By changing *Window* and *Pnum* parameters (accessible from the HatSnakeFilter plugin GUI) one can tune the filter sensitivity and number of protrusions to be removed respectively. The MeanSnakefilter on the next slot smoothens the contour using a simple running average filter (Fig. 2 and Fig. 3). Filters can be applied for each frame separately. Running automatic segmentation will apply the current configuration of filters to subsequent frames. Buttons *Populate fwd* and *Copy prev* make it possible to copy filters between frames without having to re-run the segmentation.


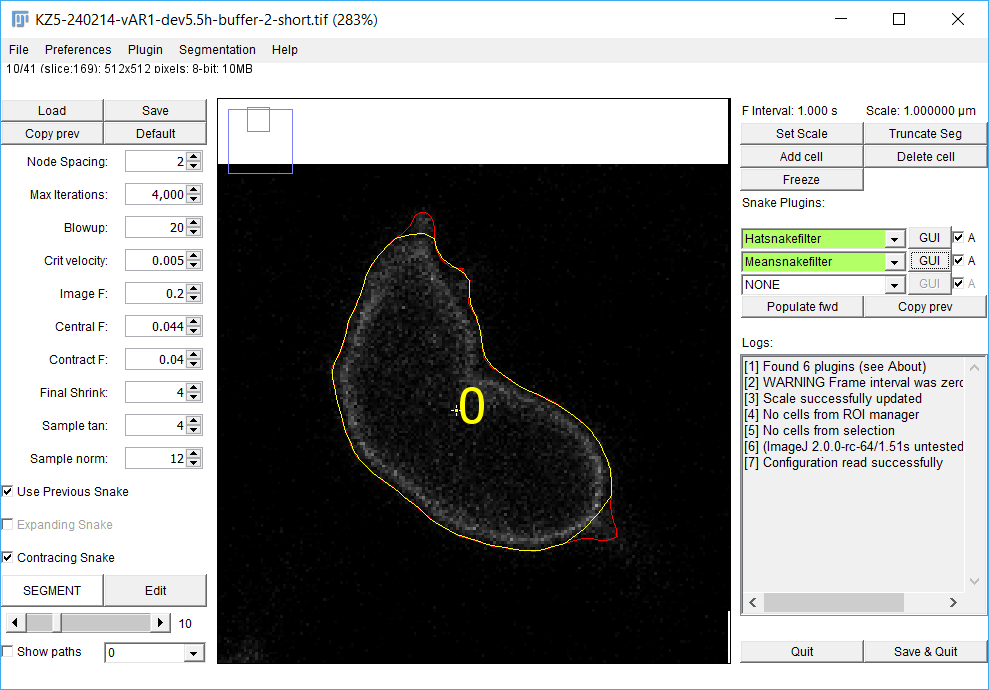


Fig. 2. Cell contour for frame 1 processed by HatSnakeFilter and then by MeanSnakeFilter – example of removing spikes. The red contour is the original one, the yellow contour is the result of filtering by filters activated in the filter stack.


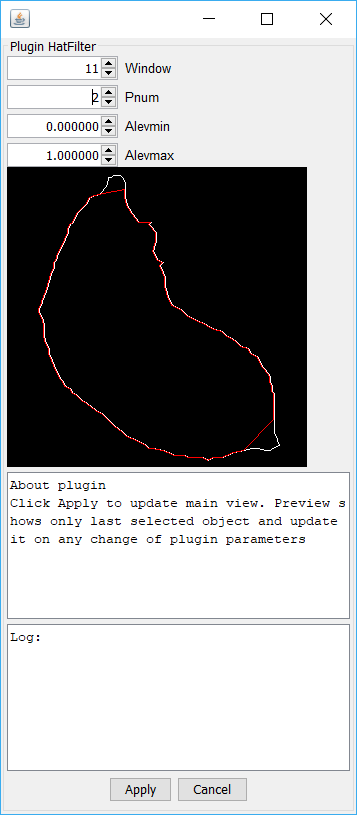

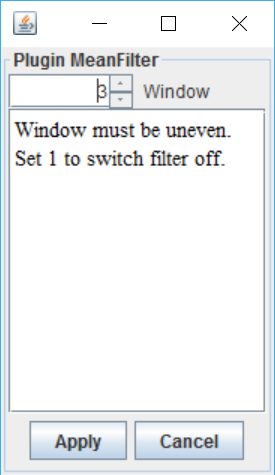


Fig. 3. HatSnakeFilter and MeanSnakeFilter windows opened by GUI button next to filter’s slot.

## Restoring contours from masks

Another useful feature implemented recently is the possibility of creating cell contours from binary or grayscale masks (background is expected to be black, 0x00). Such masks could be obtained from the QuimP Random Walk module (described in the chapter Random Walk Segmentation module) or any other segmentation method. The module also tries to track cells between adjacent frames by testing their overlapping (for binary input) or using pixels values if input mask is 8-bit grayscale image.

### Suggested workflow

1. Open *KZ5-240214-vAR1-dev5.5h-buffer-2-short.tif* image in Fiji and start BOA.
2. Go to *Segmentation->Binary Segmentation* in the BOA menu to start the plugin (Fig. 4).
3. Click *Load mask* to load *KZ5-240214-vAR1-dev5.5h-buffer-2-short_snakemask.tif* that contains a mask. One can also select any image already opened in ImageJ using the *Get Opened* selector below.
4. Select discretisation step; a step of 1 means that every pixel on the outline will be converted to a vectorised node on a contour.
5. Click *Apply* to preview results in BOA window.
6. Close the plugin window when finished.


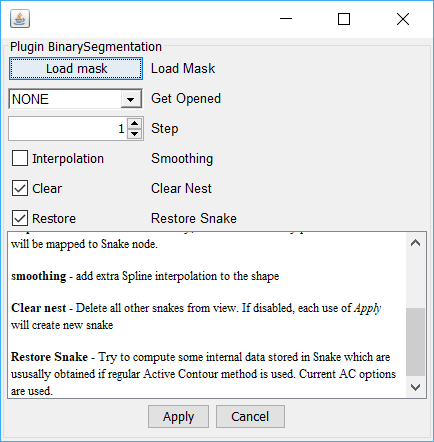


Fig. 4. Binary segmentation plugin.

The reverse operation, namely converting BOA contours to binary masks, is also possible. Refer to chapters Mask Generator and Multiple cell segmentation below for further details.

# Additional modules

There are additional plugins (modules) distributed with QuimP which have now become an integral part of it (Fig. 5) and provide new opportunities for image pre-processing and data post-processing.


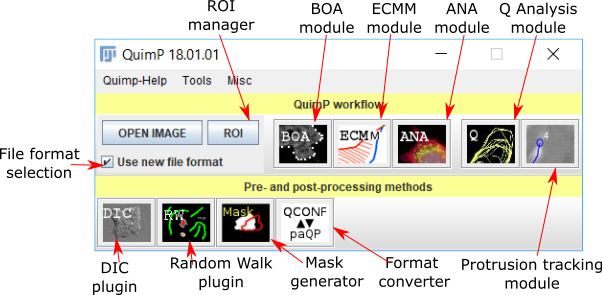


Fig. 5. QuimP toolbar with available plugins on bottom line.

Five pre- and post-processing plugins are available:

1. DIC plugin – image pre-processing plugin for reconstructing DIC^[[4]](#footnote-5)^ microscopy images.
2. Random Walk Segmentation – image pre-processing plugin implementing efficient Random Walk segmentation algorithm (Grady, 2006).
3. Mask Generator – data post-processing plugin. Allows to generate binary masks using BOA segmentation results. Essentially, it converts contours generated by BOA to images.
4. Format converter – converts between old QuimP11 data format and new QCONF. It allows also for exporting csv files.
5. Protrusion tracking module – post-processing module for protrusion analysis and visualisation (under development).

## DIC module

Differential interference contrast microscopy (DIC) enhances the contrast in unstained, transparent samples by exploiting minute differences in optical path lengths between two orthogonally polarized beams that travel through the specimen at a slightly offset position (given by the shear angle), and which are then recombined afterwards. This results in a characteristic bas-relief observed in DIC images, which makes identification of cellular structures easy for the human observer, but is the source of problems in automatic analysis of these images. Image contrast and object boundaries are well defined along the direction of the shear angle, but perpendicularly to it contrast is low and edges difficult to discern. Moreover, in the resulting DIC image strong gradients in image intensity along the shear angle negatively influence standard image processing methods like global thresholding or edge detection, producing insufficient results like discontinuous regions or edges.

The new DIC plugin in QuimP reconstructs images obtained from differential interference contrast microscopes making them more suitable for processing in the BOA module.  The local contrast of cells after reconstruction is less dependent on the direction with respect to the shear angle, and pixel intensities only take positive values. The plugin employs the algorithm described in (Kam, 1998), which works for shear angles being multiples of 45 degrees. Either single images or time lapse movies from DIC microscopy are accepted by the plugin.

### Suggested workflow

- Open provided DIC image *AX3.tif^[[5]](#footnote-6)^* in ImageJ. The algorithm (Kam, 1998) is sensitive to low-frequency intensity gradients usually observed in form of an uneven background caused for example by nonuniform backlight illumination or varying thickness of the sample. Thus, good results demand high-quality samples.
- Run DIC plugin from QuimP toolbar (Fig. 5)


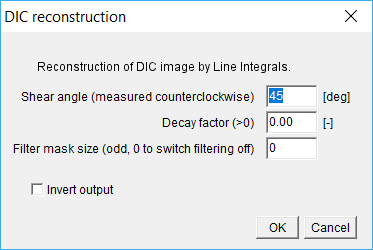


Fig. 6. DIC plugin interface.

- Set the following parameters: *Shear angle* – 45, *Decay factor* – 0.07, *Filter mask* – 3.
- Usually the DIC plugin returns inverted images – dark objects on brighter background. If that image will be further processed using the BOA module, it has to be inverted (Tick *Invert output* option in DIC plugin or use *Edit*->*Invert* from ImageJ menu).
- Select the reconstructed image and run the BOA module then draw initial ROI around cell (make sure that right tool is selected in Fiji) and click *Add cell*. Try a segmentation with the parameters shown in Fig. 7.


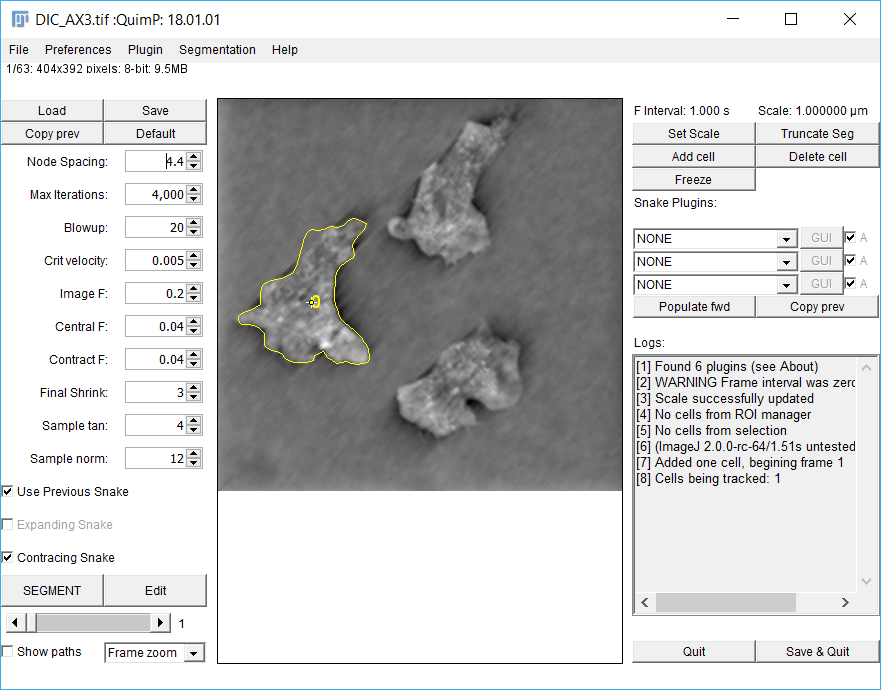


Fig. 7. Segmentation of DIC data within QuimP.

## Mask Generator module

This module can convert cell contours into binary image masks resulting in a filled white cell shape on a black background. Masks are widely used in computer graphics for processing and analysing selected regions of an image only. ImageJ is equipped with a large number of relevant procedures for working with image masks, which makes it possible to employ additional ImageJ analysis routines, if needed.

The Mask Generator plugin works with *QCONF* files saved by the BOA plugin. It is not possible to use it with the old file format (*paQP*), unless it has been converted to *QCONF* by Format Converter (available from QuimP toolbar menu). By default, the mask image is displayed on the screen and saved on disk in the same directory as the QCONF file, using the suffix *_snakemask*.

### Suggested workflow

- Run Mask Generator module from QuimP toolbar (Fig. 5).
- Point to *talA_mNeon_bleb_0pt7pctagar_FLU-11-21.QCONF* file.
- A binary mask image converted from cell contours stored previously in the QCONF file by the BOA module will be displayed on the screen and saved to disk (Fig. 8). The default location is the directory of the QCONF file that has been used. The quality of masks depends on the quality of segmentation, dimensions of the image are the same as the source image.


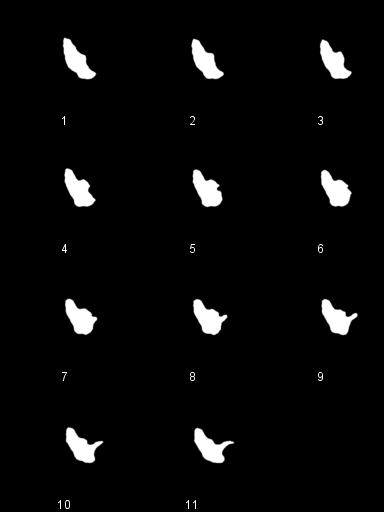

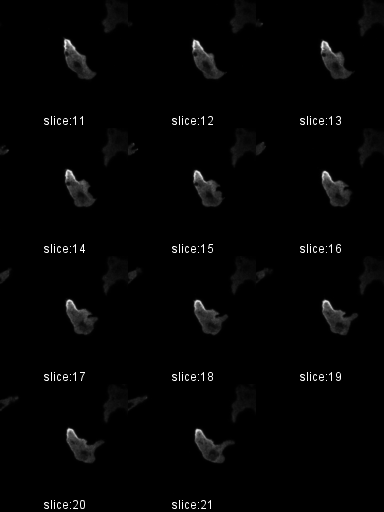


Fig. 8. Binary masks (left panel), and original datafile (right panel).

- Use *Edit->Selection->Create Selection* to convert binary masks to ImageJ ROIs. Using ImageJ’s ROI manager these ROIs can be shown on top of for example the original (unsegmented) image.

### Merging cell contours and source image

The Mask Generator plugin can be used for producing images which show the segmented cell contour on top of the original image. This feature is typically used for example to visually assess the quality of the segmentation.

- Generate binary mask as shown in previous example.
- Open the original source file in ImageJ. Normally this will be the file which has been processed in BOA module to produce the corresponding QCONF file. Following on from the previous example the original source file will be *talA_mNeon_bleb_0pt7pctagar_FLU-11-21.tif*.
- Activate the binary mask image by clicking in the window and apply *Process->Binary->Outline*
- Call *Image->Color->Merge Channels.* As red channel select the outlined image, as grey the original one. Click *Apply*. One should obtain a red cell outline on top of the source image as shown in Fig. 9.


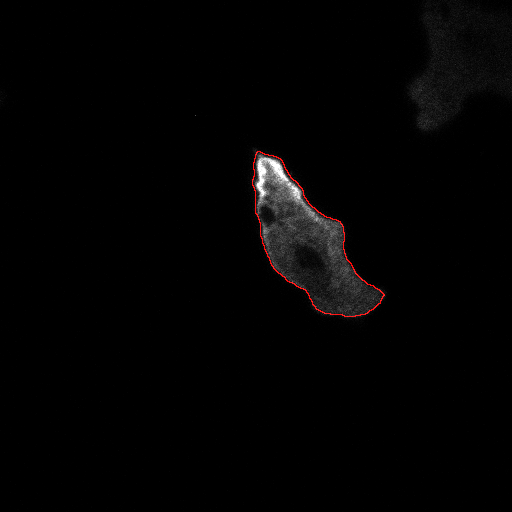


Fig. 9. Combination of cell outline and original image for one frame. Possible application of Mask Generator plugin.

## Random Walk Segmentation module

This plugin implements the Random Walk segmentation algorithm described in (Grady, 2006). It improves segmentation when working with shapes containing cavities, where active contour methods often fail. Results of the Random Walk segmentation can be imported into BOA (see chapter Restoring contours from masks) in order to produce *QCONF*/*paQP* files that can be further processed by the ECMM, ANA or QAnalyse modules.

### Suggested workflow for manually labelled images

1. Open image that will be analysed (e.g. *talA_mNeon_bleb_0pt7pctagar_FLU-11-21.tif*).
2. Open QuimP bar and select Random Walk module.
3. Choose your image in *Image* *selection*.
4. Prepare seed image that contains preliminarily labelled foreground and background regions. The seeds can either consist of a scribbled RGB image or binary masks. Here, we will label the source image using build-in tools:
   1. Select *Create image* in *Get seeds from* frame.
   2. Select first slice in your original image and click *Clone* to duplicate it and convert it to RGB format. Pick the *Current slice* option. The seed image can have the same number of slices as the source image or only one slice. In the first case the user would need to label each individual slice. Otherwise, the Random Walk plugin will generate seeds for next slice automatically, using the currently segmented frame after processing it by the method selected in *Inter-process* frame. (see Fig. 10).
   3. Make sure that correct image is selected in *Seed build* selector.
   4. Use BG and FG buttons to select background and foreground pen respectively. Scribble background and foreground objects (or use provided *seeds.tif* image).

- Set parameters - default values are usually fine.
  1. Select *CONTOUR* in *Shrink method*
- Select *Show seeds* to inspect seed propagation when segmentation is done and *Show preview* to observe progress.
- Click *Run*. The segmented image will appear when the process is finished.


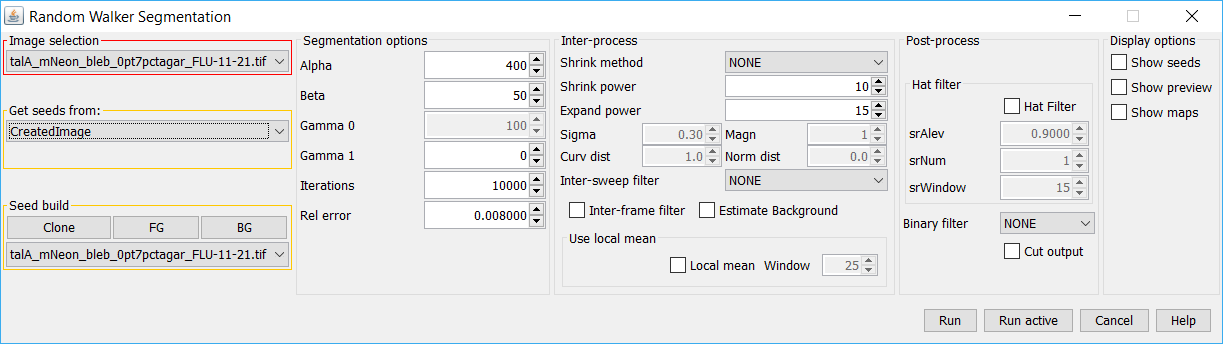


Fig. 10. Random Walk plugin window and seed propagation stack.

### Multiple cell segmentation

The Random walk module is able to segment and track multiple cells preserving their boundaries when they touch each other. It is possible to track up to 255 objects. Example workflow:

1. Open example image *Stack-30.tif* in ImageJ.
2. Open QuimP toolbar and run Random walk module.
3. Select your image in *Image selection.*
4. In *Get seeds from* select *Rois*. This tool allows you to label objects in the image.
5. Click *Clone* below and clone first frame from the image – a new image should appear.
6. Click *Seed* next to *Clone*, it opens a new tool together with the ROI manager window.
   1. Scribble objects in the image clicking *New FG* button each time when you want to add the current selection as a label of a new object. You can also label the same object using several separated ROI objects. In order to do so, first add each ROI to ROI Manager by using *Add* button from ROI Manager window, then click *New FG* button to finalise labelling.
   2. Then label some background around cells and click *New BG*.
   3. Click *Finish* at the end.
   4. All labels are stored as Rois in the ROI manager. You can also load those already prepared:
      1. Open *Seed* tool again and clear ROI manager.
      2. In ROI manager click *More*->*Open* and select provided file *RoiSet-Stack-30-6cells.zip.*
      3. You can use ImageJ tools to visualise loaded Rois.
      4. Click *Finish* at the end.
7. You should see information about the number of labelled objects below the *Seed* button in Random Walk module.
8. Open console from Fiji->Window->Console to track progress and relative error. Set parameters as shown in Fig. 11 and click *Run*.


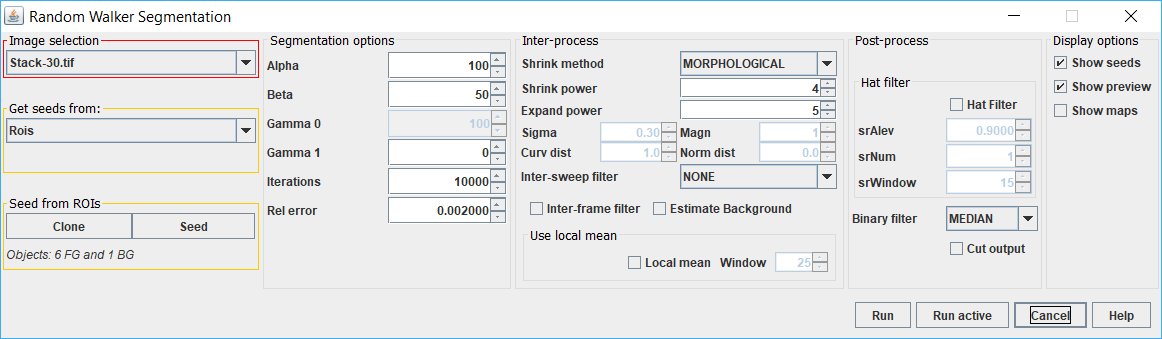


Fig. 11. Proposed settings for segmentation of Stack-30 tif sequence with labels specified in RoiSet-Stack-30-6cells.zip file.

Each cell is plotted in different colours, unique across the sequence (Fig. 12). This format is recognised and accepted by Binary segmentation plugin (chapter Restoring contours from masks). This approach helps segmenting touching cells which is usually difficult using active contour segmentation. Moreover, it allows to apply the QuimP workflow to images that could not be successfully segmented by BOA module. Either the proposed Random walk module or any other software can be used for this purpose.


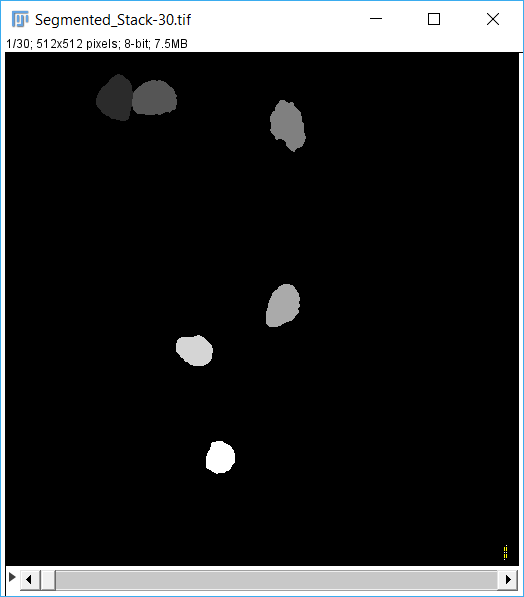

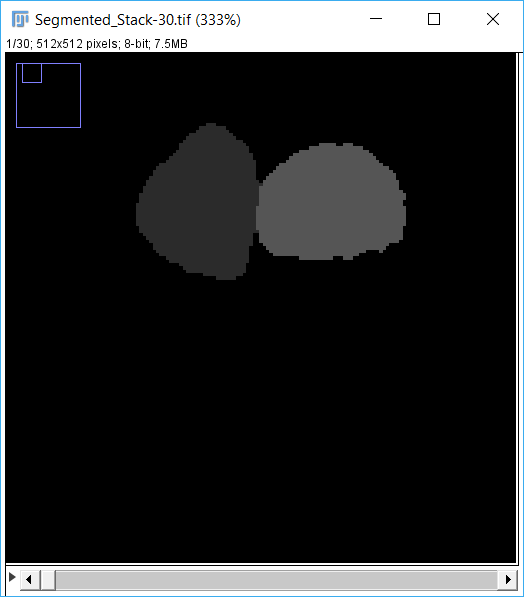


Fig. 12. First frame of segmented sequence Stack-30.tif (Segmented_Stack-30.tif included in supplementary data). Right image shows touching cells which have been correctly separated.

### Combined segmentation - utilize active contours for seed generation, and local mean feature

The Random walk segmentation plugin works best when combining it with the active contour method from the BOA plugin to generate seeds automatically. This approach allows to deal with difficult cases that contain concave regions in the cell contour and strong gradients in fluorescence. The active contour segmentation is used for generating preliminary results utilising the user-friendliness of the BOA module and having the advantage of being very fast. The random walk method is then used for obtaining a more refined segmentation of the cell edge that better preserves details. The BOA module and the Random Walk module can exchange data through binary masks, thus the final segmentation can be imported back into BOA for further analysis within a typical QuimP workflow. Here is a recipe how one can use this feature:

1. Open the image that will be analysed (e.g. *talA_mNeon_bleb_0pt7pcagar_FLU.tif* from AcRw folder).
2. Open QuimP bar and select BOA module.
   1. Select a cell and perform an active contour segmentation using safe parameters (default are fine) clicking *Add cell* button. Segmentation may not be accurate. It is not necessary to get a precise representation of cell shape at this stage.
   2. Exit the BOA module saving *QCONF* file.
3. Open Random walk module from QuimP toolbar
   1. Choose your image in O*riginal image* selector.
   2. Select *QCONF file* in *Get seeds from* frame and load file produced by BOA module in previous step.
   3. Set parameters as shown in Fig. 13.
   4. *Run* segmentation
4. After successful segmentation import masks into the BOA plugin as described in paragraph Restoring contours from masks. Obtained results usually are better than those from active contour or random walk when manual seeding is used.


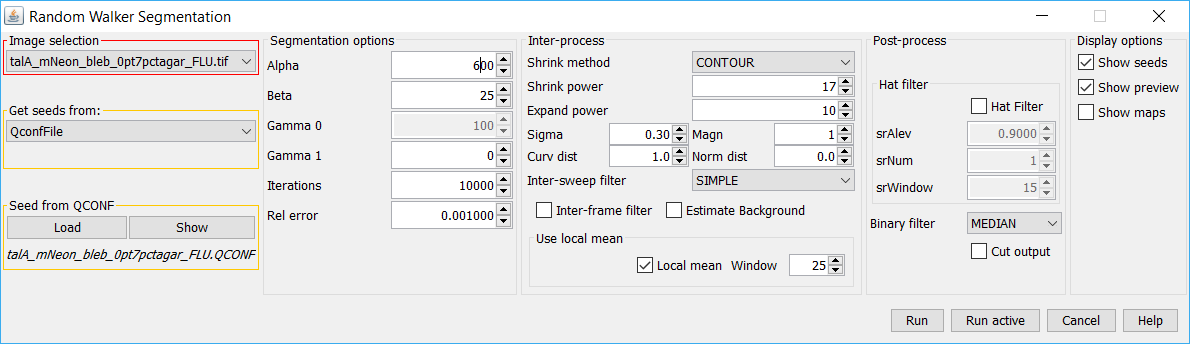


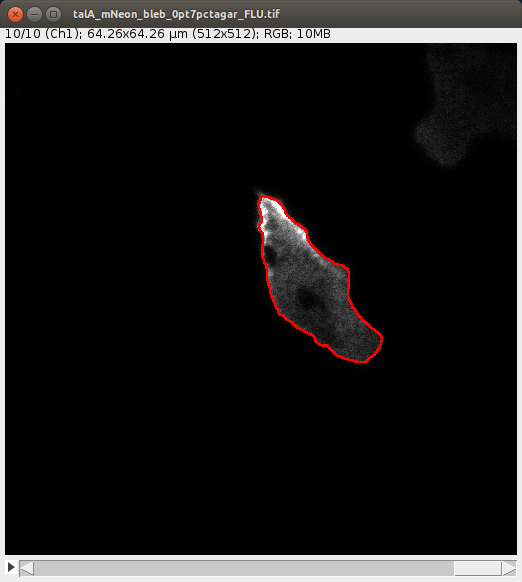


Fig. 13. Suggested segmentation parameters for exemplary talA_mNeon_bleb_0pt7pcagar_FLU.tif file (upper) and result of segmentation (lower).

### Combined segmentation - use Random walk segmentation directly from BOA module

Random walk segmentation generally deals better with cavities but on the other hand it is slower than the active contour method and has a tendency to under-segment regions with strong intensity gradients (as discussed in supplementary material SI-C). QuimP2018 offers a unique combination of the random walk and the active contour method, which has been integrated into the BOA module. The random walk module is available as a BOA plugin and it can be called interactively by users for particular frames only, similarly to other plugins described in section Working with plugins - possible use case. For example, consider the following workflow:

1. Open in Fiji *talA_mNeon_bleb_0pt7pcagar_FLU-11-21.tif* image.
2. Run BOA module from QuimP Toolbar or from *Plugins* menu.
3. Set segmentation parameters as in Fig. 14.
4. Select cell (make sure that selection tool is chosen in Fiji) and click *Add cell* in BOA. Then click *Segment* (or you can load already segmented configuration *talA_mNeon_bleb_0pt7pcagar_FLU-11-21.QCONF*)
5. Go to frame 7 – there is a cavity where Active Contour method failed. Select *Randomwalksnakefilter* in filter stack (Fig. 15), click GUI button to open filter configuration and set options as shown in Fig. 15. Click Apply (note that the code is not optimised yet and segmentation is relatively slow, one can track progress in Fiji, Window->Console)
6. You should obtain the result shown in Fig. 16. The yellow line is the newly segmented shape, whereas the original unprocessed outline is shown in red. You can apply *Meansnakefilter* to obtain a smoother outline.
7. Repeating steps 4-6 for each wrongly segmented frame one can significantly improve the overall quality of the segmentation. *Copy prev* and *Populate fwd* buttons help in transferring the current configuration between frames. Upon clicking *Segment* the current configuration will be applied to all subsequent frames.


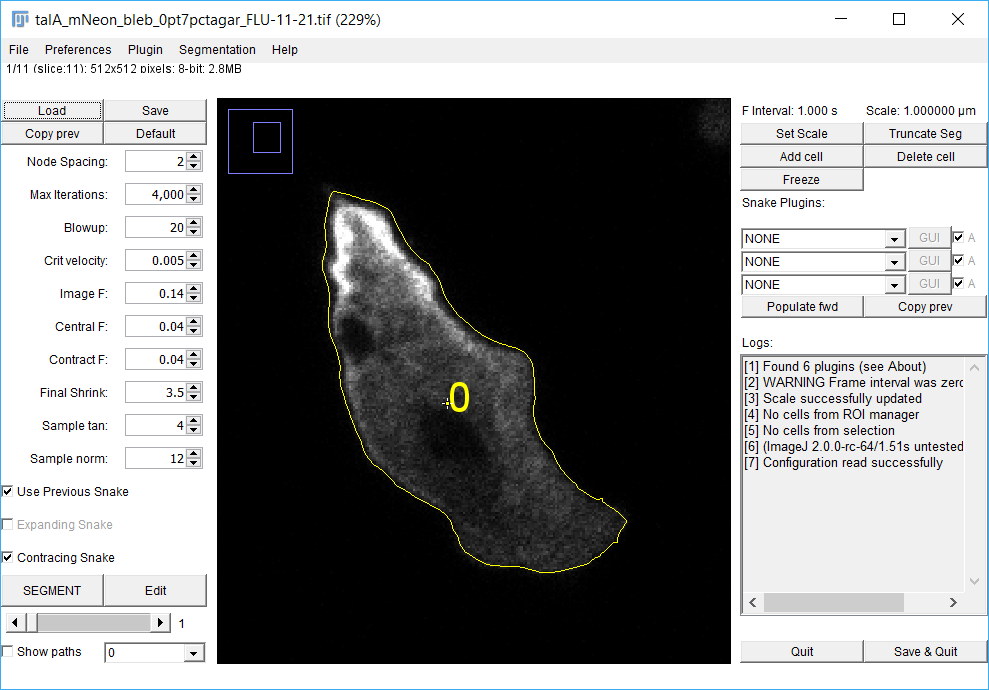


Fig. 14. Active contour segmentation parameters.


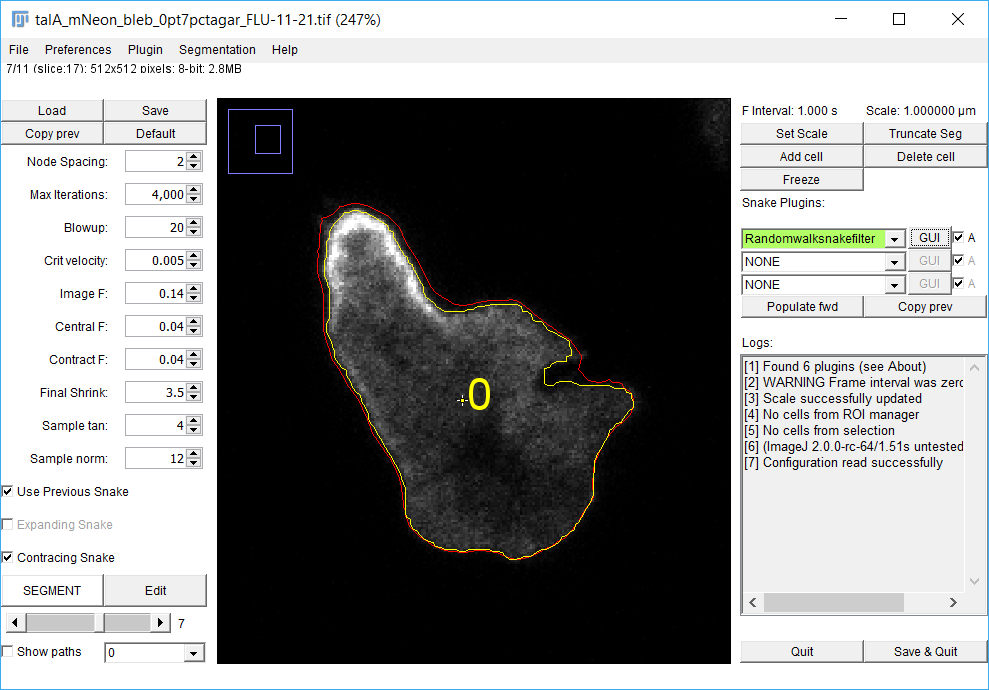

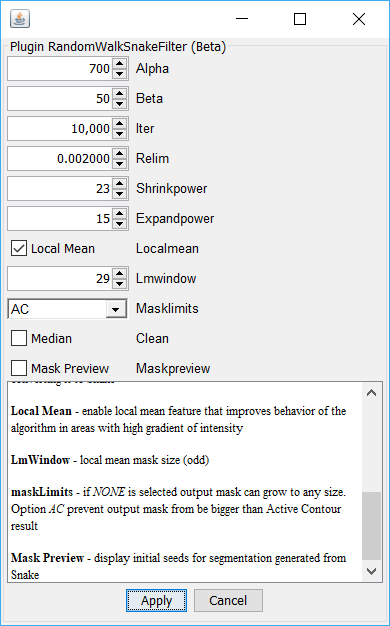


Fig. 15. BOA module on left with selected Random walk filter. Random walk filter parameters (on right).


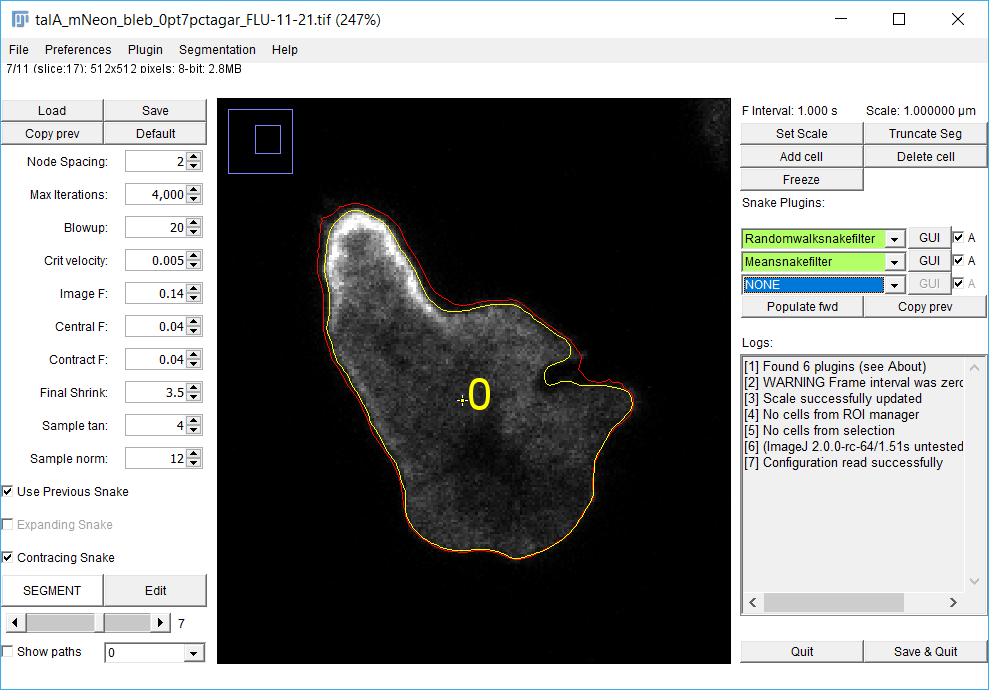


Fig. 16. Result of combined Active Contour and Random walk segmentation in BOA.

# References

Grady,L. (2006) Random walks for image segmentation. *IEEE T Pattern Anal,* **28**(11), 1768–1783.

Kam,Z. (1998) Microscopic differential interference contrast image processing by line integration (LID) and deconvolution. *Bioimaging,* **6**(4), 166–176.

1. <http://warwick.ac.uk/quimpapi> [↑](#footnote-ref-2)
2. Data kindly provided by Evgeny Zatulovskiy/Rob Kay, MRC-LMB Cambridge [↑](#footnote-ref-3)
3. Filters must be installed in the Fiji plugin directory to be accessible from BOA. All filters mentioned here are included in the default QuimP installation. [↑](#footnote-ref-4)
4. Differential Interference Contrast [↑](#footnote-ref-5)
5. The example data has been kindly provided by Rob Insall, Beatson Institute, Glasgow. [↑](#footnote-ref-6)
