# Supplementary material for: QuimP: analyzing transmembrane signalling in highly deformable cells
Source: Bioinformatics. 2018 Mar 16;34(15):2695–7. doi: 10.1093/bioinformatics/bty169 (PMC6061833; doi:10.1093/bioinformatics/bty169)
Supplement: Supplementary Data [file bty169_supp.zip › bty169-suppl_data/Supplementary_material_B.pdf]

Supplementary material B – QuimP API

This supplementary material briefly describes the new QCONF file format and QuimP's Application Programming Interface (API).

Test data and example files can be downloaded from http://www.warwick.ac.uk/quimp/test_data/.

Contents

INTRODUCTION TO THE QCONF FILE FORMAT 2

CONVERSION BETWEEN FORMATS 2
DEVELOPER API AND QCONF IN DETAIL 2
QCONF AND MATLAB 3

Introduction to the QCONF file format

QuimP now uses the human-readable JSON format for storing parameters and data in *QCONF* files, which are initially created by the BOA module. Subsequent modules (ECMM, ANA, QA) extend the *QCONF* container by module specific data like e.g. algorithm parameters, computed results, etc., so that the *QCONF* file contains a complete record of the entire workflow. The new format is fully portable (JSON parsers are available for all leading programming languages) and easy to reuse, share or archive.

Conversion between formats

To maintain backwards compatibility, all modules that were available in QuimP11 still support the previous file format (**.paQP*). It is also possible to convert between the two formats using the Format Converter available from the QuimP Toolbar either under Tools menu or as separate module (Fig. 1). More details can be found in the User Manual (http://warwick.ac.uk/quimpdoc).

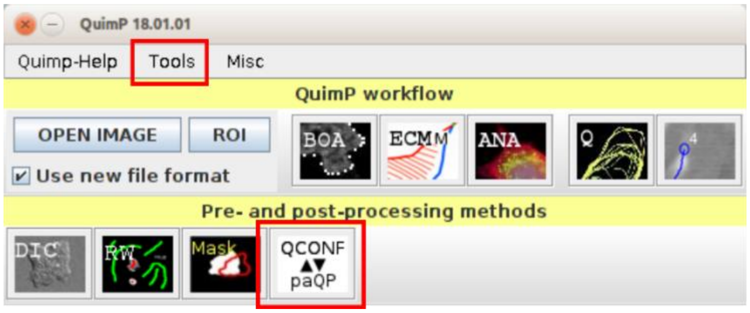

Fig. 1. Format converter available from QuimP toolbar.

Developer API and QCONF in detail

A high-level application programming interface (API) delivered with QuimP makes it possible to load and process data from QCONF files as well as to extend QuimP by new modules. The provided time series example *talA_mNeon_bleb_0pt7pctagar_FLU.QCONF* contains segmentation results that have been created by the BOA module. Since the JSON file format is human-readable and self-descriptive, this file can be opened and viewed/modified in a standard text editor. *Key: value* pairs are encapsulated in larger structures by curly brackets. Basic knowledge of Java or another objective language will help to fully understand the *QCONF* structure. The best place to learn about the *QCONF* format are the QuimP Java API reference pages (http://warwick.ac.uk/quimpapi).

A good starting point for understanding the *QCONF* structure is the `DataContainer` class that defines the skeleton of the format[1]. For example, the `BOAState` field contains the whole configuration of the BOA module, in particular the configuration of the active contour algorithm, given as an array of `BOAState.SegParam` objects (the configuration is stored for every frame separately). For a single frame the relevant part of the *QCONF* file looks like follows:

```
"segParamSnapshots": [

        {
          "nodeRes": 6.0,
          "blowup": 20,
```

---

[1] In the enclosed file some fields could be missing, because they might require that some specific other modules are run to create them.

```
            "vel_crit": 0.005,
            "f_central": 0.04,
            "f_image": 0.14,
            "max_iterations": 4000,
            "sample_tan": 4,
            "sample_norm": 12,
            "f_contract": 0.04,
            "finalShrink": 3.0,
            "use_previous_snake": true,
            "showPaths": false,
            "expandSnake": false,
            "min_dist": 6.0,
            "max_dist": 11.4
          },
```

Listing 1. Fragment of QCONF file with parameters of the Active Contour method for one frame.

The key-value pairs match segmentation parameters available from the BOA user interface. Other data, e.g. cell outlines are stored inside the `Nest` structure that contains an array of `SnakeHandlers` objects that hold most important parameters of the nodes of cell outlines (class `Snake` and related super-classes).

QCONF and Matlab

QCONF files can be imported into Matlab through JSON parsers like the free JSONlab, which converts the data to regular structures and arrays. Part of the JSON file from Listing 1 imported into Matlab looks like:

```
j.obj.BOAState.segParamSnapshots

ans =

  1×10 cell array

    [1×1 struct]    [1×1 struct]    [1×1 struct]    [1×1 struct]    [1×1 struct]    [1×1
struct]    [1×1 struct]    [1×1 struct]    [1×1 struct]    [1×1 struct]

j.obj.BOAState.segParamSnapshots{1}

ans =

  struct with fields:

               nodeRes: 6
                blowup: 20
              vel_crit: 0.0050
             f_central: 0.0400
               f_image: 0.1400
        max_iterations: 4000
            sample_tan: 4
           sample_norm: 12
            f_contract: 0.0400
           finalShrink: 3
    use_previous_snake: 1
             showPaths: 0
           expandSnake: 0
              min_dist: 6
              max_dist: 11.4000
```

The attached file *talA_mNeon_bleb_0pt7pctagar_FLU.mat* is the result of the *QCONF* file being imported by JSONlab. The script *plotoutlines.m* is a simple example of post-processing cell contours in Matlab. Refer to the code and comments for details.

```
% Demonstrate accessing data in QCONF file imported to Matlab
% Plot cell outlines across frames on one plot.

% QCONF file was converted to Matlab format via JSONlab
load 'talA_mNeon_bleb_0pt7pctagar_FLU.mat'

% There is only one cell, so go directly to the array of Snakes
finalSnakes = j.obj.BOAState.nest.sHs{1,1}.finalSnakes;
% get number of frames
fn = length(finalSnakes);
% generate color scale
color = summer(fn+1);

figure;
hold on
% iterate over frames and plot outlines
for f = 1:fn
    % get number of nodes for Snake at frame f
    nn = length(finalSnakes{f}.Elements);
    % collect all x,y coordinates in arrays by iterating over nodes
    x = [];
    y = [];
    for c = 1:nn
        node = finalSnakes{f}.Elements{c};
        x = [x node.point.x];
        y = [y node.point.y];
    end
    % close shape
    x = [x x(1)];
    y = [y y(1)];
    % plot
    plot(x,y,'color',color(f,:));
end
axis square
grid on
xlabel('Screen coordinate, X')
ylabel('Screen coordinate, Y')
title('Cell outlines across time')
```

Listing 2. Listing of plotoutlines.m routine.

The result of running *plotoutlines.m* is shown in Fig. 2.

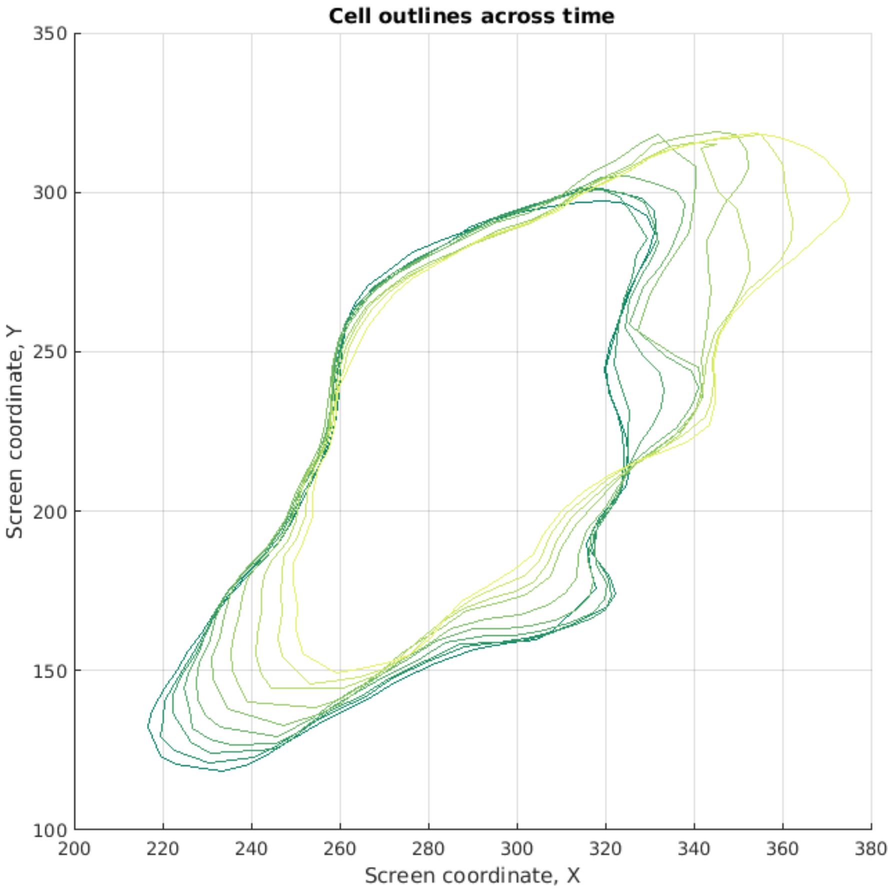

Fig. 2. Cell outlines produced by plotoutlines.m script from QCONF file converted to Matlab structures.
