# Supplementary material for: QuimP: analyzing transmembrane signalling in highly deformable cells
Source: Bioinformatics. 2018 Mar 16;34(15):2695–7. doi: 10.1093/bioinformatics/bty169 (PMC6061833; doi:10.1093/bioinformatics/bty169)
Supplement: Supplementary Data [file bty169_supp.zip › bty169-suppl_data/Supplementary_material_C.docx]

Supplementary material C – Segmentation quality assessment

This supplementary material contains an extended analysis of segmentation accuracy of the Active Contour and Random Walk methods implemented in QuimP.

Test data and example files can be downloaded from <http://www.warwick.ac.uk/quimp/test_data/>.

Contents

[Experimental datasets 2](#_Toc506194566)

[Data segmentation 3](#_Toc506194567)

[Quality assessments 4](#_Toc506194568)

[References 18](#_Toc506194569)

# Experimental datasets

We used several experimental datasets to evaluate the quality of segmentation offered by QuimP2018.

**TalA-mNeon blebbing cells (2% and 0.7%), experimental sets A, B, C and D:**

*Dictyostelium* talin A-/B- double knockout cells, transformed with talin A-mNeon, a FERM domain protein which links the cell membrane to the actin cortex. Cells are forced to chemotax under a thin agarose overlay of 0.7% or 2% concentration. The cells migrate using either actin driven pseudopodia, hydrostatic pressure driven blebs, or a combination of the two. TalA-mNeon fluorescence is seen to be asymmetrical, with enrichment of talin A at the cell rear.

Files included in supplementary materials:

- C1-talA_mNeon_2pct_bleb_Image39_FLU.tif (**A**)
- C1-talA_mNeon_2pct_bleb_Image40.tif (**B** and **C**)
- C1-talA_mNeon_bleb_0pt7pctagar_FLU.tif (**D**)

**TalA-GFP randomly migrating cells, experimental set E:**

*Dictyostelium* talin A-/B- double knockout cells, transformed with talin A-GFP. Developed cells are randomly migrating in low fluorescence buffer.

File included in supplementary materials:

- C1-talA_GFP_rnd_motility_FLU.tif (**E**)

**Actin randomly migrating cells, experimental set F:**

*Dictyostelium* Ax2 cells expressing ABD-GFP, an F-actin marker. Developed cells are randomly migrating in low fluorescence buffer, using actin-driven pseudopodia.

File included in supplementary materials:

- July09ABD_GFP_actin_rnd_motility.tif (**F**)

**Actin cells under agar, experimental set G:**

*Dictyostelium* Ax2 cells expressing ABD-GFP. Cells are forced to chemotax under a thin agarose overlay of 1% concentration. The cells migrate using both, pseudopodia and blebs.

File included in supplementary materials:

- July14ABD_GFP_actin_1pctagar.tif (**G**)

All images were recorded on a Zeiss 710 confocal microscope at 2fps with a 63x oil emersion objective. Subsequently, they were processed completing the entire QuimP2018 workflow (<http://warwick.ac.uk/quimpdoc>) using three segmentation methods; standard active contour algorithm implemented in the BOA module, the random walk algorithm that is available as a plugin of QuimP2018, followed by binary segmentation performed in BOA and Trainable Weka Segmentation available as ImageJ plugin (Arganda-Carreras *et al*., 2017). The algorithms were denoted by suffixes *ac*, *rw* and *we* which have been added to the name of experimental datasets.

# Data segmentation

Data were segmented in the BOA module (Active Contour method) and the Random Walk module separately. In both approaches we were interested in getting a good segmentation with minimal effort. Thus, we tuned segmentation parameters so that we were able to obtain a good overall segmentation of the entire sequence, without manually tuning individual frames, where segmentation might have been less successful.

We used our modified Random Walk algorithm that utilizes a rough segmentation from the Active Contour method as initial state. The whole process consists of four main steps: 1) rough active contour segmentation, 2) contour shrinking to obtain automated seeds for the random walk method 3) refined random walk segmentation and 4) shape filtering. The first step was performed in the BOA module using fail-safe parameters, whereas steps 2 to 4 are implemented in the Random Walk module (<http://warwick.ac.uk/quimpdoc>).

For the sake of comparison with other leading segmentation tool, we processed our data with the Trainable Weka Segmentation method available as ImageJ plugin (Arganda-Carreras *et al*., 2017). We have experimentally preselected a set of features that seemed to produce the best results and then processed all our samples using the same configuration settings depicted in Fig. 1. The classifier was trained separately for each dataset on three frames from the beginning, middle and end of the sequence, providing initial seeds manually. Output masks were cleaned from isolated pixels and other objects separated from cells being within a region of interest (this aspect is discussed further below).


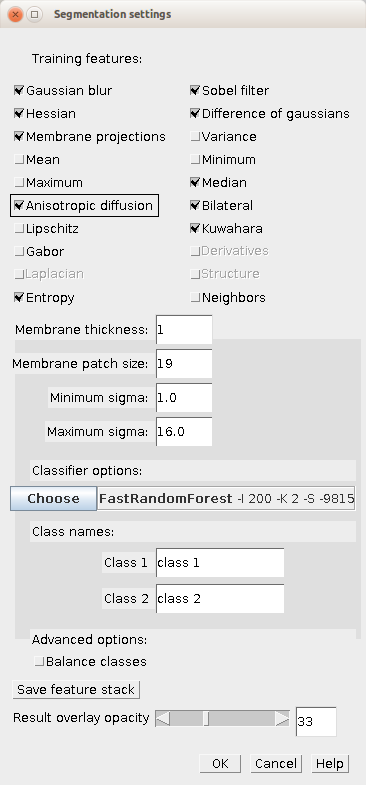


Fig. 1. Configuration of Weka classifier used to segment the example data. Anisotropic diffusion turned out to be most influential feature, which is interesting as anisotropic diffusion is directly related to the random walk method used in QuimP3.

# Quality assessments

A modified Random Walk method shows improvements in critical areas (white arrows) where either Active Contour segmentation or Random Walk segmentation alone usually fail because of concavities and strong intensity differences in the cortex. Selected results which demonstrate the high efficiency of our proposed modification of the Random Walk algorithm are shown in Fig. 2.


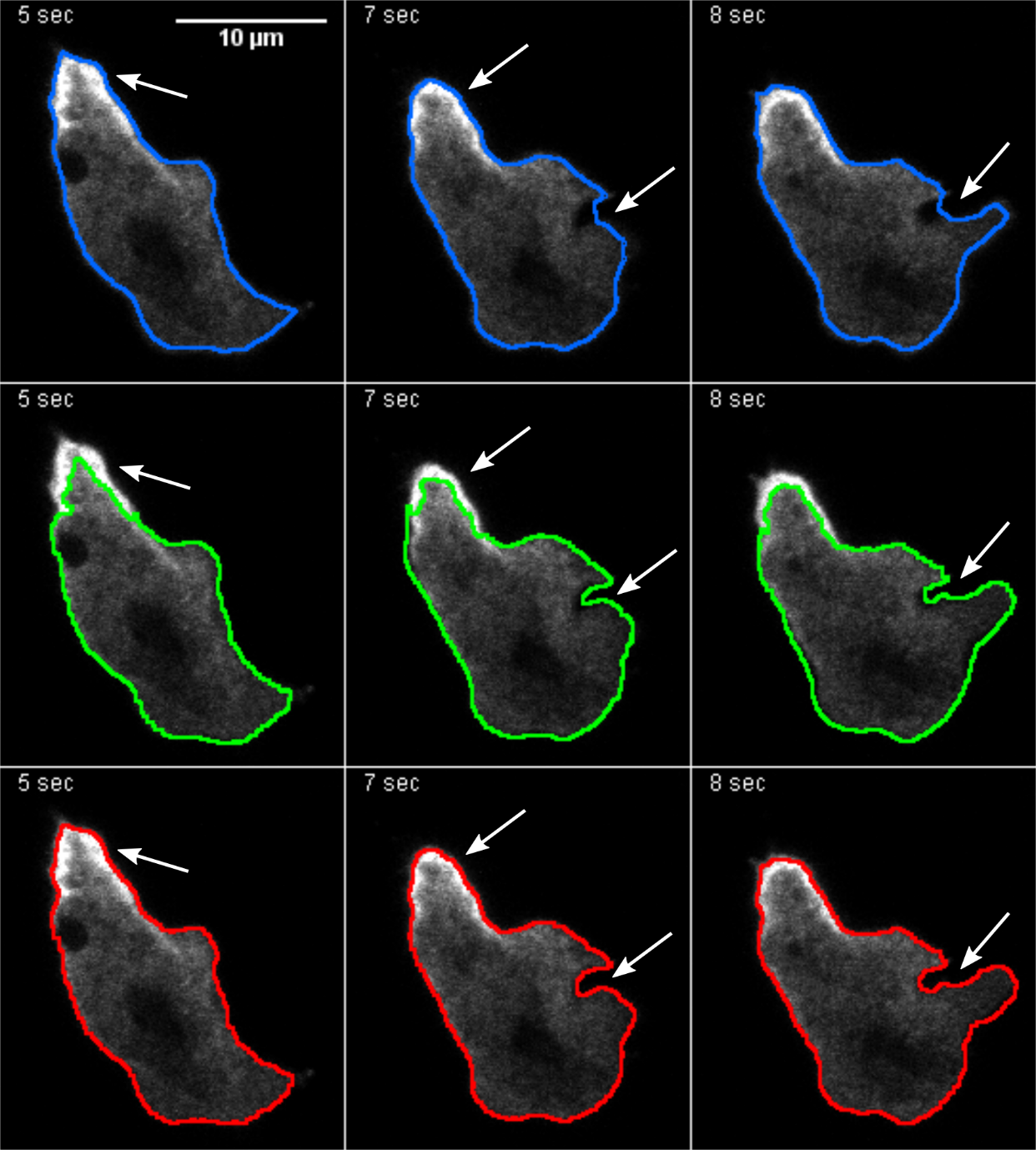


Fig. 2. Typical problems in cell segmentation when using Active Contours (blue outline) or a conventional Random Walk method (green). Arrows point to difficult regions. Our modified Random Walk method (red) solves some of these issues. Pixels that are part of the outline belong to the segmented object. Vesicles close to the cell boundary as seen in the 5 sec frame usually are also difficult to segment. A new filter based on local circularity is however able to remedy such cases efficiently.

We assessed the quality of QuimP segmentation, computing and analysing statistics derived from the confusion matrix. Quantifying cortical fluorescence in the ANA module, or tracking boundary markers over time, or analysing protrusions, all require well segmented cell boundaries, and as such is certainly the most important aspect in cell motility analysis. Typical metrics, commonly used for evaluation of the quality of segmentation, rely on pixelwise binary comparison of a gold standard with the test sample obtained from a segmentation algorithm. Small but crucial differences located on the cell boundary may not be caught with adequate sensitivity. Therefore, the Hausdorff metric *d_H_* (Hausdorff, 1914) seems to be a more appropriate metric for assessing the image segmentation in cell motility analysis applications. The Hausdorff distance is defined as the maximum distance of a point on a reference cell outline to the nearest point on the segmented outline. This distance should be shorter than the cortex width used to define the cortical region within which fluorescence intensities are typically sampled (the parameter cortex width is configured in the ANA module). However, the Hausdorff distance may not reflect the true effort a user has to make to manually tune the segmentation result as it is only considering the maximum distance between the sample and the gold standard, so that two different sample segmentations might give the same *d_H_* even if one of them has a much closer fit on average.

Therefore another, more informative metric we introduced is the ratio of the number of false positive, *FPL* or false negative pixels to the boundary length *l_c_* which exhibits enhanced sensitivity regarding small differences between cell outlines.

$$FPL=\frac{\sum FP}{l_{C}}$$

$$FNL=\frac{\sum FN}{l_{C}}$$

Additionally, we have used accuracy , F1 score metric and the Rand index (Rand, 1971) as quality metrics

$$ACC=\frac{TP+TN}{P+N}$$

$$F1=\frac{2TP}{2TP+FP+FN}$$

where

$$\begin{matrix} P=TP+FN \\ N=FP+TN \end{matrix}$$

*TP* in the equations above stands for the number of true positive pixels (correctly classified as foreground), *TN* is the number of true negative pixels (classified as background). *FP* and *FN* are false positive and negative pixels, respectively.

Plots below show Hausdorff distances computed for each frame of every sequence. Pictures on the right show the frame from sequence with maximum *d_H_*. Cumulative results are presented in Table 1.

| 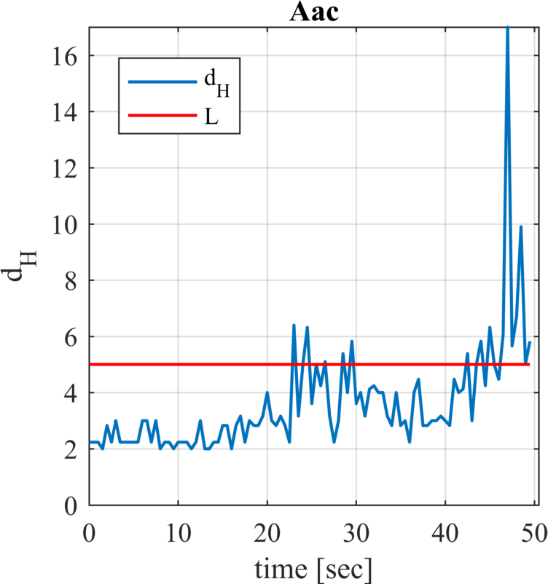 | 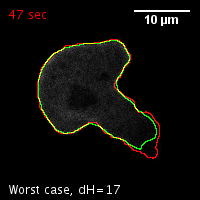 |
| --- | --- |
| 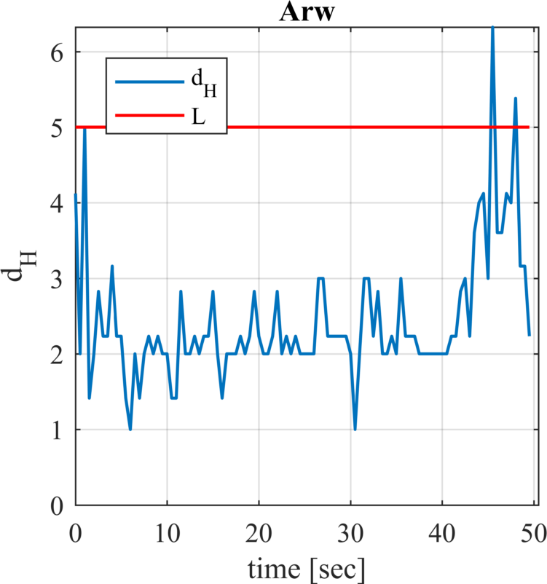 | 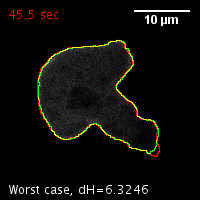 |
| 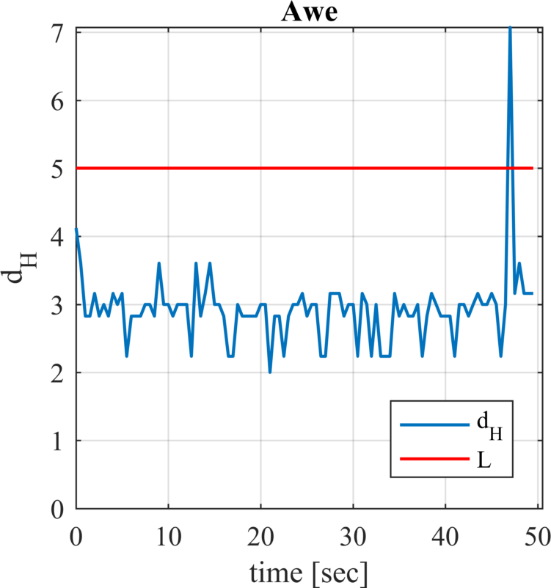 | 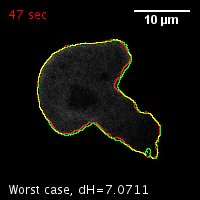 |

Fig. 3. Hausdorff distances for each frame for active contour (ac), random walk (rw) and trainable Weka (we) method for case A. Pictures on the right show the reference outlines (gold standard in red) versus the actual segmentations (in green) for the corresponding frames with maximal Hausdorff distance (worst case)

| 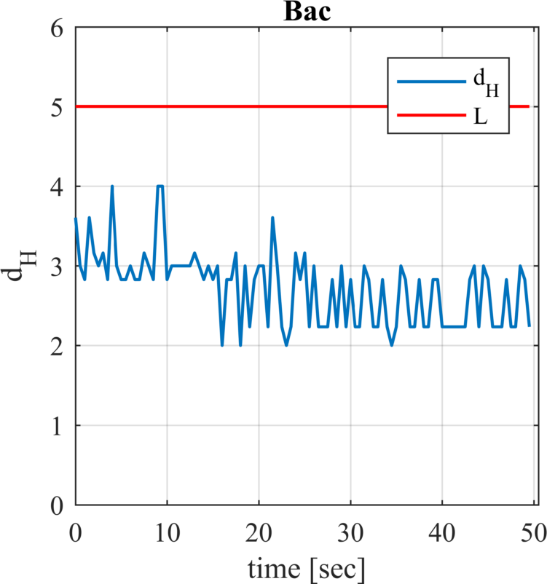 | 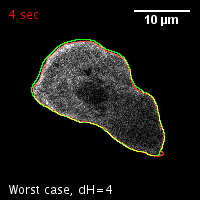 |
| --- | --- |
| 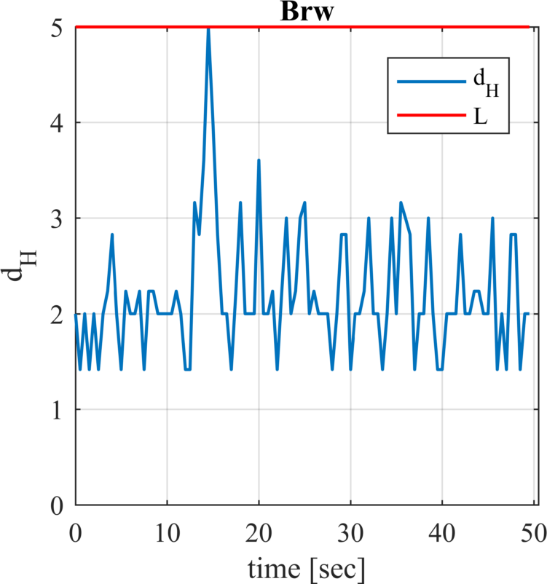 | 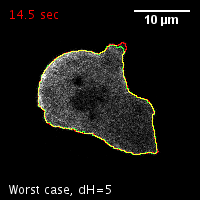 |
| 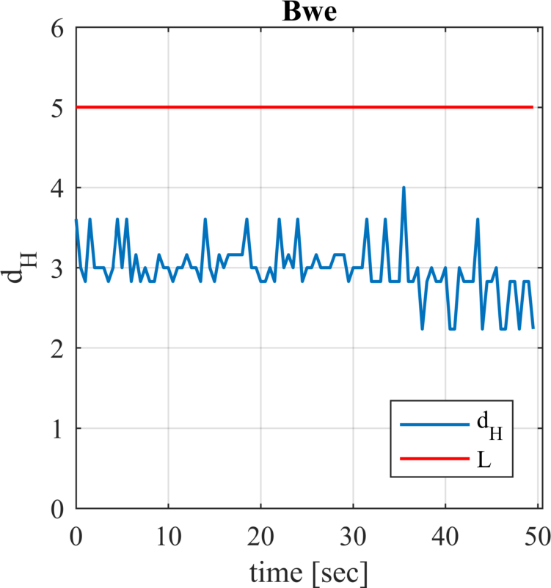 | 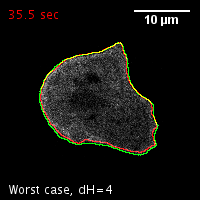 |

Fig. 4. Hausdorff distance for each frame for active contour (ac), random walk (rw) and trainable Weka (we) method for case B. Pictures on the right show the reference outlines (gold standard in red) versus the actual segmentations (in green) for the corresponding frames with maximal Hausdorff distance (worst case)

| 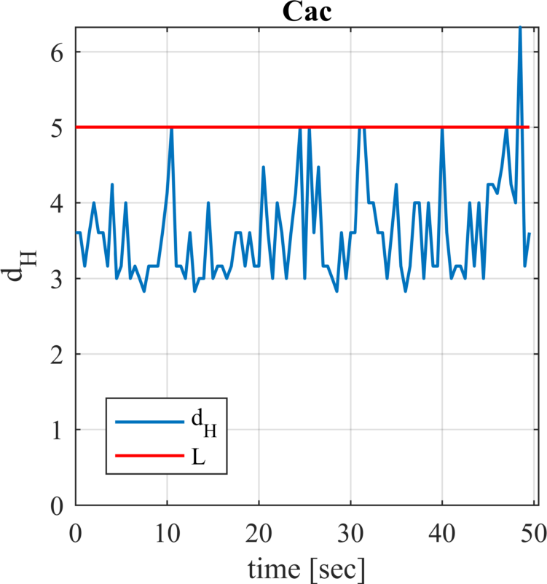 | 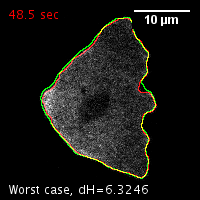 |
| --- | --- |
| 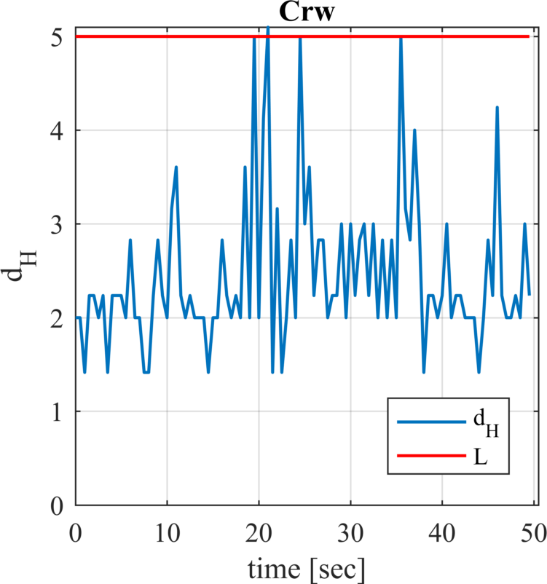 | 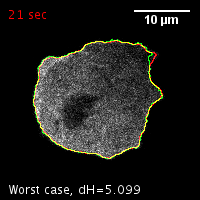 |
| 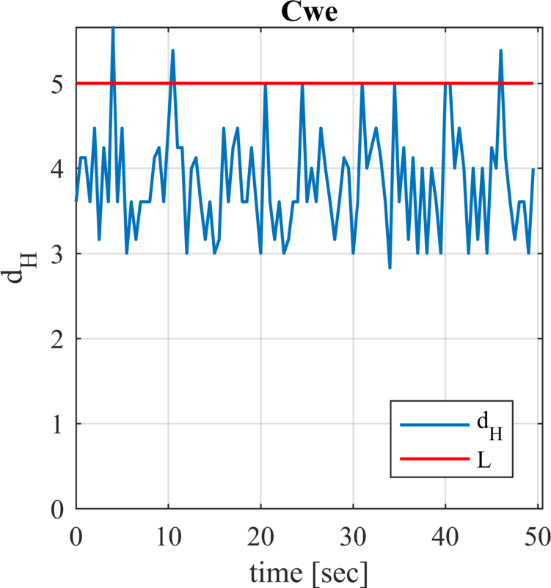 | 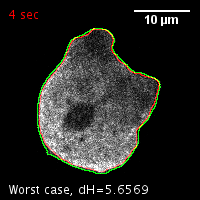 |

Fig. 5. Hausdorff distance for each frame for active contour (ac), random walk (rw) and trainable Weka (we) method for case C. Pictures on the right show the reference outlines (gold standard in red) versus the actual segmentations (in green) for the corresponding frames with maximal Hausdorff distance (worst case).

| 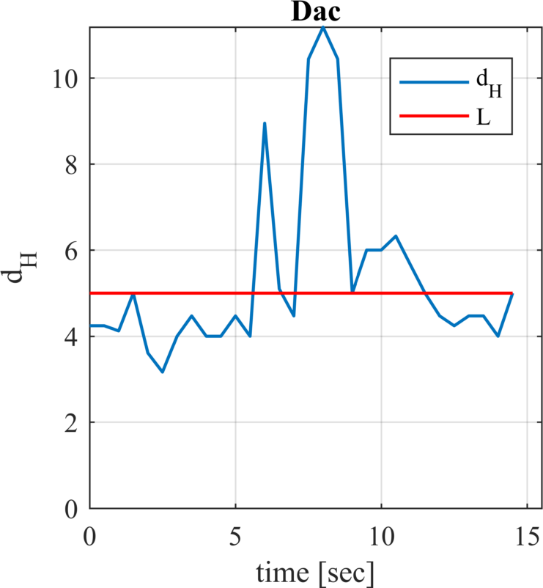 | 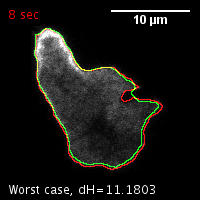 |
| --- | --- |
| 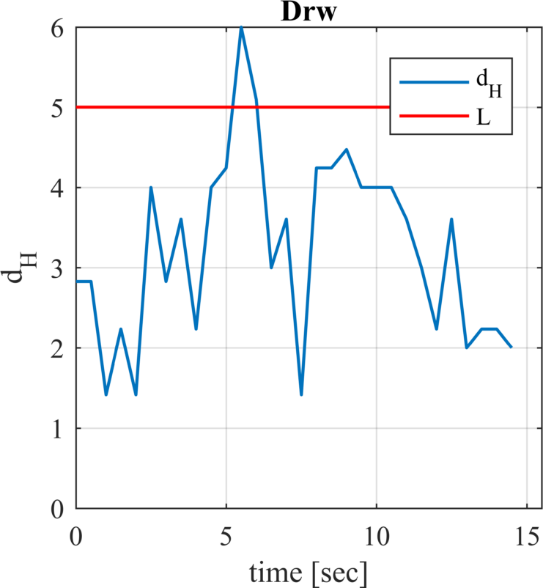 | 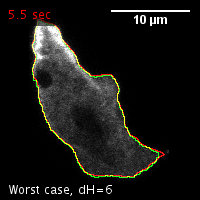 |
| 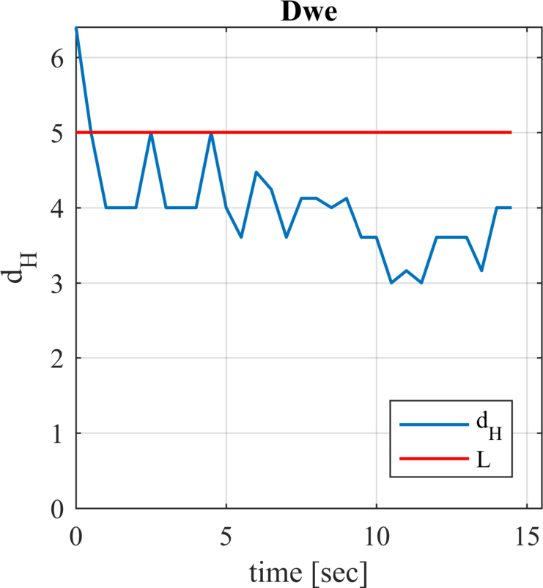 | 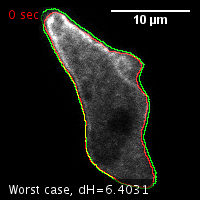 |

Fig. 6. Hausdorff distance for each frame for active contour (ac), random walk (rw) and trainable Weka (we) method for case D. Pictures on the right show the reference outlines (gold standard in red) versus the actual segmentations (in green) for the corresponding frames with maximal Hausdorff distance (worst case)

| 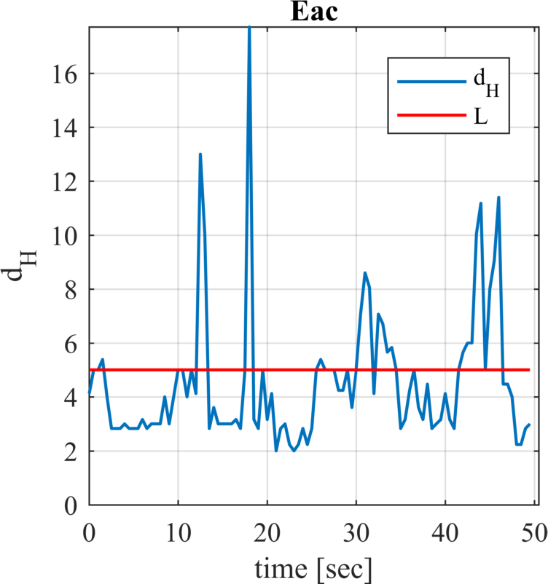 | 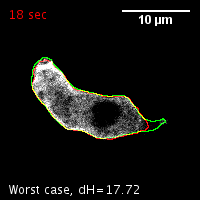 |
| --- | --- |
| 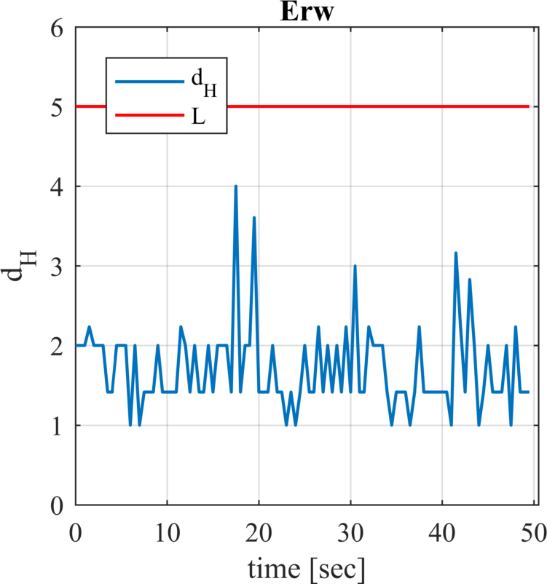 | 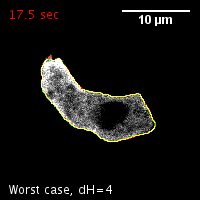 |
| 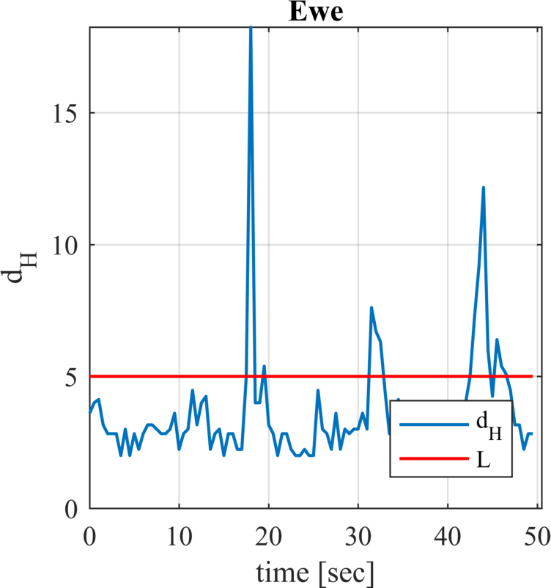 | 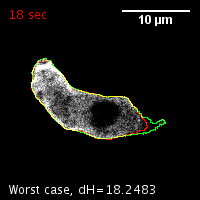 |

Fig. 7. Hausdorff distance for each frame for active contour (ac), random walk (rw) and trainable Weka (we) method for case E. Pictures on the right show the reference outlines (gold standard in red) versus the actual segmentations (in green) for the corresponding frames with maximal Hausdorff distance (worst case)

| 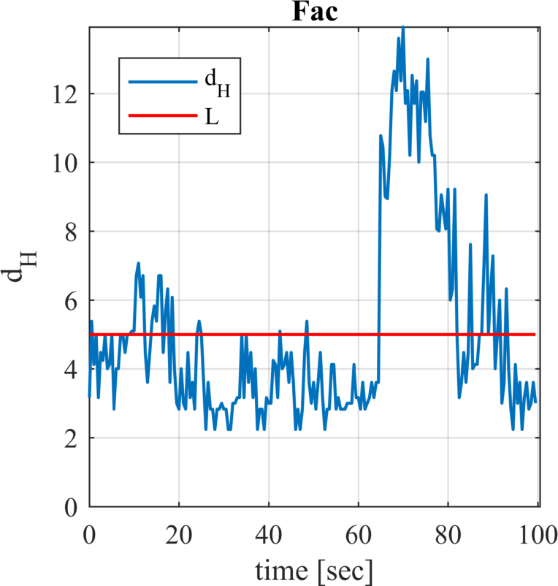 | 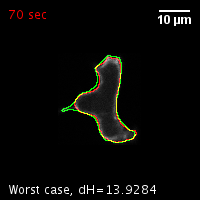 |
| --- | --- |
| 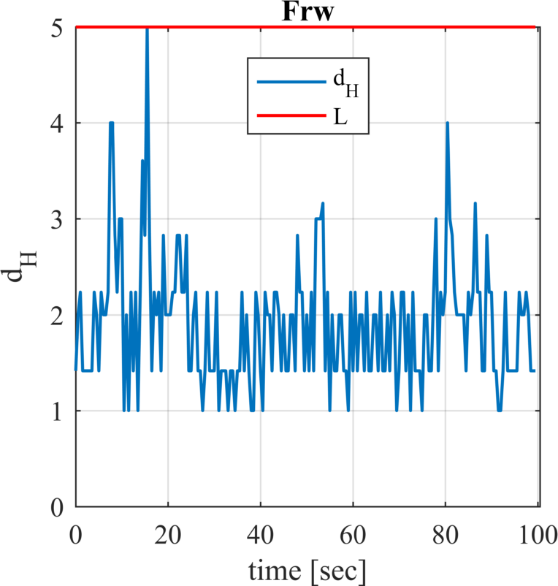 | 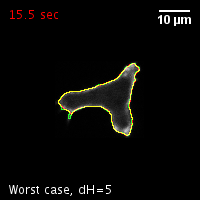 |
| 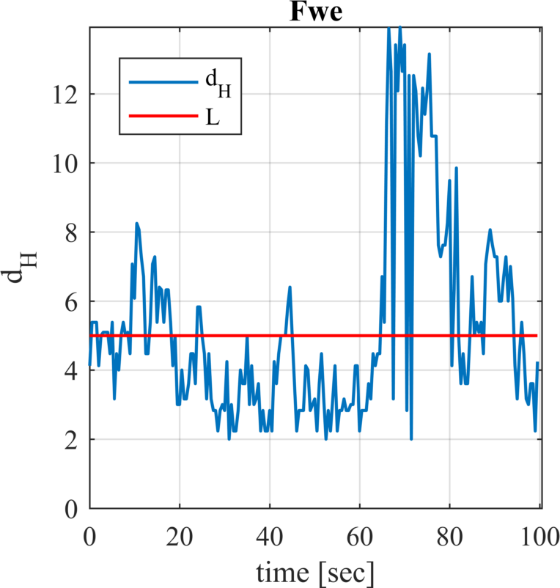 | 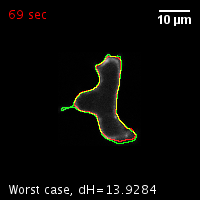 |

Fig. 8. Hausdorff distance for each frame for active contour (ac), random walk (rw) and trainable Weka (we) method for case F. Pictures on the right show the reference outlines (gold standard in red) versus the actual segmentations (in green) for the corresponding frames with maximal Hausdorff distance (worst case)

| 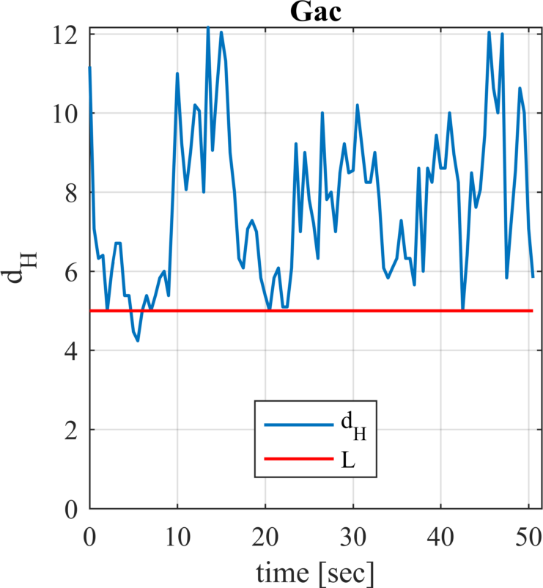 | 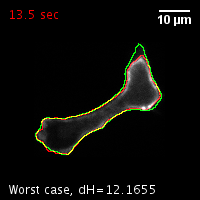 |
| --- | --- |
| 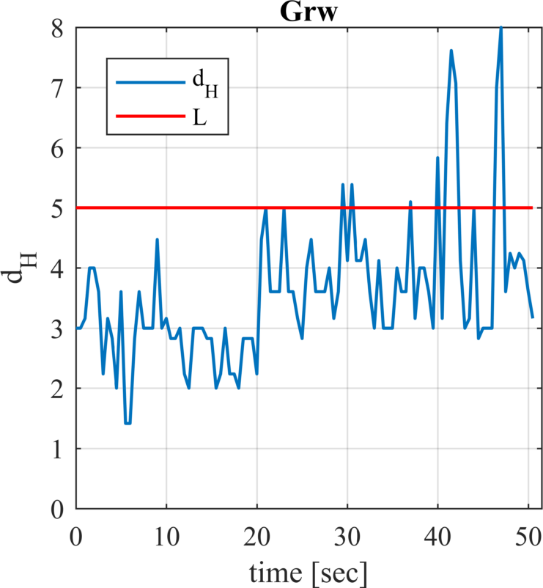 | 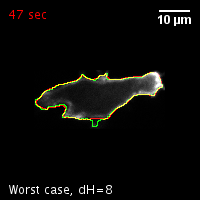 |
| 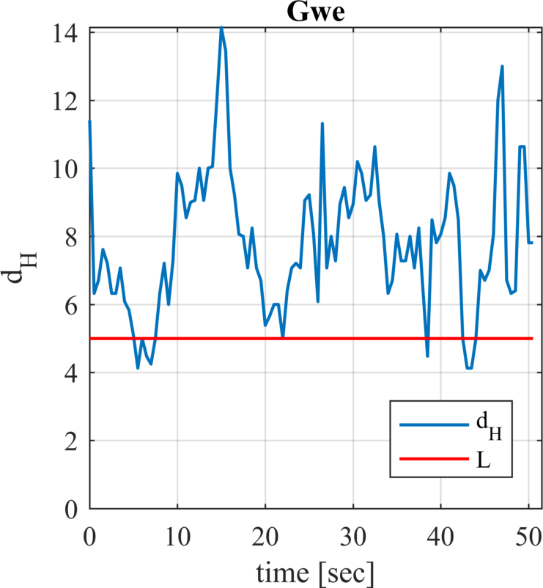 | 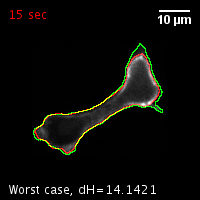 |

Fig. 9. Hausdorff distance for each frame for active contour (ac), random walk (rw) and trainable Weka (we) method for case G. Pictures on the right show the reference outlines (gold standard in red) versus the actual segmentations (in green) for the corresponding frames with maximal Hausdorff distance (worst case)

The Random Walk segmentation method definitely better copes with concave regions of the cell outline. Usually, such sections of the cell contour are not segmented correctly by the active contour method due to high local curvature values. In contrary, the RW method often fails to properly segment protrusions that are weakly connected with the main cell body. The Weka method produces results comparable to our RW algorithm, but it has tendency to slightly over-segment in some cases. The FNL value is much lower here than for Random Walk method (Fig. 10), but comes at the prize of a worse FPL value. It must be emphasised that the Weka classifier is pixel-based whereas combined AC-RW QuimP method is related to objects. Thus the latter will only consider pixels preliminary selected by AC method as those belonging to the cell region. On the contrary, Weka assigns to the same class all pixels with similar properties over the entire image, capturing sometimes unwanted artefacts such as other cells or background noise.

Table 1 summarises the results previously presented in Fig. 3 - Fig. 9. In most cases segmentation can be considered as overall successful, with a limited number of frames that do not meet the level of acceptance, which we have set at a Haussdorff distance of *d_H_*=5. Above this distance local readouts of fluorescent in the cortex will be compromised. Usually, insufficient segmentation is observed for shapes with strong local curvature in concave regions. Table 1 shows that the Random Walk segmentation significantly increases the number of acceptable frames.

Table 1. Number of frames where d_H_ meets the acceptance criteria.

| **Case** | **Number of accepted frames/Total number of frames** | | |
| --- | --- | --- | --- |
|  | ***Active Contour*** | ***Random Walk*** | ***Trainable Weka*** |
| **A** | 86 / 100 | 98 / 100 | 99 / 100 |
| **B** | 100 / 100 | 100 / 100 | 100 / 100 |
| **C** | 99 / 100 | 99 / 100 | 97 / 100 |
| **D** | 21 / 30 | 28 / 30 | 29 / 100 |
| **E** | 80 / 100 | 100 / 100 | 88 / 100 |
| **F** | 141 / 200 | 200 / 200 | 123 / 200 |
| **G** | 7 / 102 | 93 / 102 | 11 / 102 |

The following figures show distributions of the considered metrics *d_H_*, *FPL* and *FNL* computed for all frames of each example sequence.


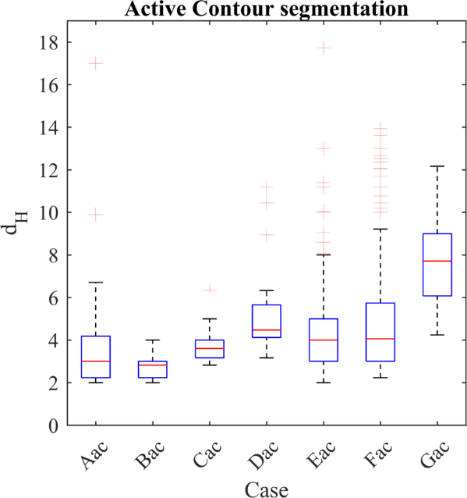

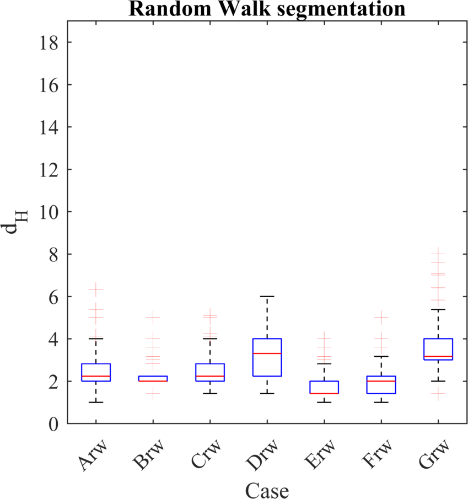

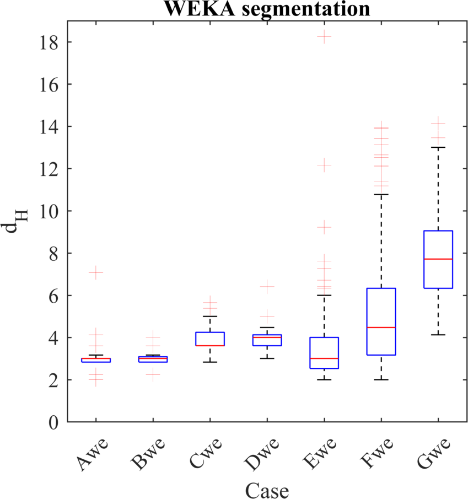


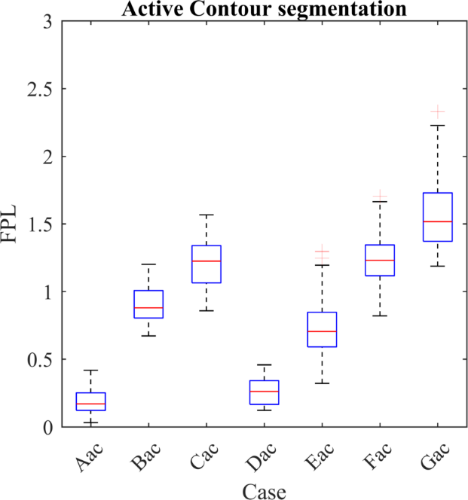

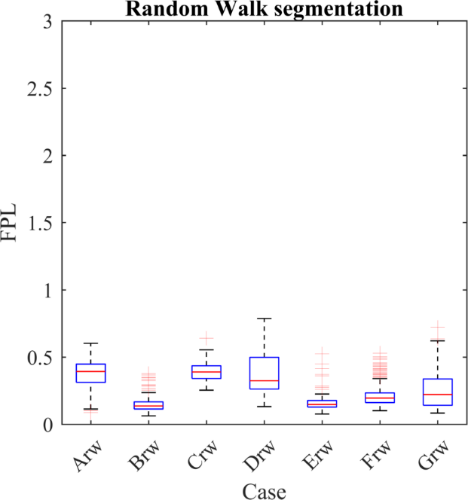

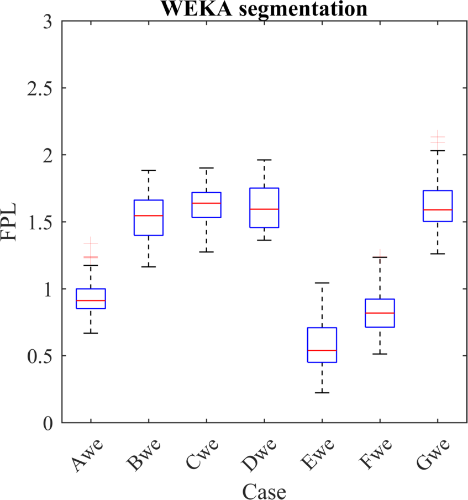


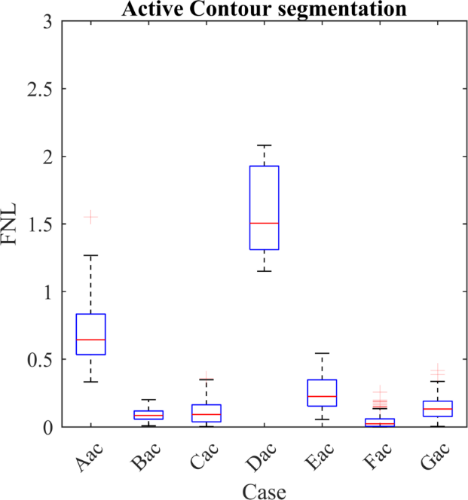

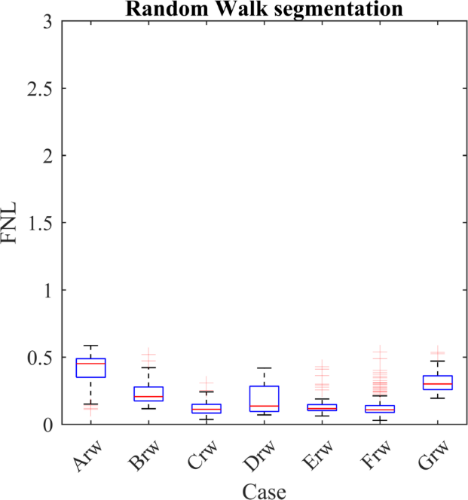

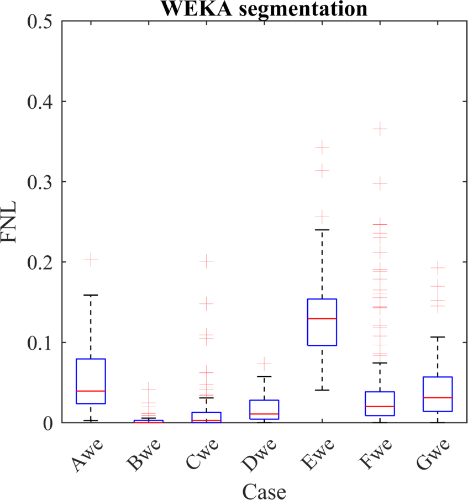


Fig. 10. Medians of selected metrics (red lines). The bottom and top of the box are the first and third quartiles, whiskers are the lowest datum still within 1.5 IQR of the lower quartile, and the highest datum still within 1.5 IQR of the upper quartile. Red crosses denote points that are beyond the 1.5*IQR range. Note that FNL values for Weka segmentation are on average lower than those for the two other methods. However, FPL and FNL values for the random walk method are consistently low across all experiments.

Kolmogorov-Smirnov test statistics showed that the test metrics do not follow a standard normal distribution (α=0.05). Table 2 summarises the median values for each test metric.

Table 2. Medians of selected metrics calculated for all frames of each example sequence.

| **Case** | **F1** | | | **Hausdorff** | | | **FPL** | | | **FNL** | | |
| --- | --- | --- | --- | --- | --- | --- | --- | --- | --- | --- | --- | --- |
|  | ***AC*** | ***RW*** | ***WE*** | ***AC*** | ***RW*** | ***WE*** | ***AC*** | ***RW*** | ***WE*** | ***AC*** | ***RW*** | ***WE*** |
| **A** | 0.98 | 0.98 | 0.98 | 3.00 | 2.24 | 3.00 | 0.17 | 0.39 | 0.91 | 0.64 | 0.45 | 0.04 |
| **B** | 0.98 | 0.99 | 0.97 | 2.83 | 2.00 | 3.00 | 0.88 | 0.14 | 1.54 | 0.08 | 0.21 | 0.00 |
| **C** | 0.98 | 0.99 | 0.97 | 3.61 | 2.20 | 3.61 | 1.22 | 0.39 | 1.64 | 0.09 | 0.11 | 0.00 |
| **D** | 0.96 | 0.99 | 0.97 | 4.47 | 3.30 | 4.00 | 0.26 | 0.32 | 1.59 | 1.5 | 0.13 | 0.01 |
| **E** | 0.97 | 0.99 | 0.98 | 4.00 | 1.41 | 3.00 | 0.70 | 0.15 | 0.54 | 0.22 | 0.12 | 0.13 |
| **F** | 0.94 | 0.99 | 0.96 | 4.06 | 2.00 | 4.47 | 1.23 | 0.20 | 0.82 | 0.02 | 0.11 | 0.02 |
| **G** | 0.94 | 0.98 | 0.94 | 7.71 | 3.16 | 7.71 | 1.51 | 0.22 | 1.59 | 0.13 | 0.30 | 0.03 |

Accuracy ACC and Rand index are not included in Table 2 as they did not discriminate enough (all close to 1.0 with differences at 5^th^ decimal place) between the active contour, random walk and trainable Weka segmentations. In contrary, the Haussdorff distance, and FPL and FNL metrics are more informative.

The statistical significance of paired AC-RW results for each metric was tested with a two-sided Wilcoxon test (Table 3) with H0 hypothesis that differences between pairs follow a distribution with zero-median.

Table 3. p-values obtained from Wilcoxon test for AC-RW pairs.

| **Case** | **Hausdorff** | **FPL** | **FNL** |
| --- | --- | --- | --- |
| **A** | 6.59e-12 | 1.08e-13 | 3.88e-16 |
| **B** | 6.43e-09 | 3.89e-18 | 4.13e-18 |
| **C** | 1.76e-14 | 3.89e-18 | 0.270 |
| **D** | 1.51e-05 | 0.037 | 1.73e-06 |
| **E** | 5.58e-18 | 3.89e-18 | 2.62e-10 |
| **F** | 4.21e-34 | 1.43e-34 | 5.22e-29 |
| **G** | 1.82e-18 | 1.82e-18 | 1.59e-16 |

Summarizing the results shown in Fig. 3 - Fig. 10 and Table 1 - Table 2 we can say that the new Random Walk module offers better segmentation quality and preserves more details than QuimP’s original Active Contour method. That can be confirmed by smaller Hausdorff distances for each test case (Fig. 10). Finally, the need for manual corrections of cell outlines in long time series can be reduced when using the RW method (Table 1). Higher FPL coefficients observed for most of AC examples is related to catching small but sometimes unwanted details like e.g. filopodia, a known problem with the active contour algorithm. On the other hand, the random walk algorithm results in slightly worse FNL measures which is a result of under-segmentation around regions with strong intensity gradients.

The quality of segmentation offered by QuimP is comparable with the Weka method even outranking it in some cases. Importantly, QuimP requires no time to generate training data like Weka does. Furthermore, potential users benefit from integration of the proposed Random Walk method in the QuimP workflow that allow them to correct particular frames interactively, stopping/resuming the process and more. Binary masks obtained by means of any other segmentation method can be easily imported and processed (including manual corrections). QuimP is also less memory hungry than Weka plugin. The real memory size allocated by Weka during processing 200 frames length F dataset was 6.3 GB whereas QuimP used only 1.3 GB^[[1]](#footnote-2)^.

# References

Arganda-Carreras,I. *et al.* (2017) Trainable Weka Segmentation: a machine learning tool for microscopy pixel classification. *Bioinformatics*, **33**(15), 2424-2426.

Hausdorff,F. (1914) *Grundzüge der Mengenlehre.* Leipzig: AMS Chelsea Publishing (reprinted).

Rand,W.M. (1971) Objective criteria for the evaluation of clustering methods. *J Am Stat Assoc,* **66**(336), 846-850.

1. RSS, measured for the whole ImageJ instance on 16-Core Linux server, all cores were utilized by Weka during segmentation. ImageJ was restarted between tests to clean up any cached data. [↑](#footnote-ref-2)
