# Supplementary material for: QuimP: analyzing transmembrane signalling in highly deformable cells
Source: Bioinformatics. 2018 Mar 16;34(15):2695–7. doi: 10.1093/bioinformatics/bty169 (PMC6061833; doi:10.1093/bioinformatics/bty169)
Supplement: Supplementary Data [file bty169_supp.zip › bty169-suppl_data/Supplementary_material_D.pdf]

Supplementary material D – Description of the modified Random Walk method

This supplementary material describes the Random Walk method implemented in QuimP.

Contents

MODIFIED RANDOM WALK METHOD **2**

MATHEMATICAL APPROACH 2

Stability of solution *2*

Computing probabilities and getting final segmentation *3*

ALGORITHM IMPROVEMENTS 4

Local mean feature *4*

Auto generating seed pixels *5*

Two pass segmentation *6*

REFERENCES **7**

Modified Random Walk method

We improved the Random Walk method, developed by (Grady, 2006) to obtain better accuracy in very inhomogeneous regions and concave regions. The main improvement is the use of local means versus global means which makes the method locally adapt to e.g. strong gradients along the cell axis. We have also implemented a contour filtering algorithm, applied in the post-processing stage, that deals with vesicles located near the cell edge, which otherwise can be improperly assigned to background.

Mathematical approach

The random walk method is a supervised image segmentation method, where the user preliminary labels foreground and background areas. It can easily be understood in terms of an anisotropic diffusion process where pixels labelled as foreground act as source (probability *P* of being foreground equals one) and pixels labelled as background (probability of being foreground equals zero) as sink. The diffusion process is inversely proportional to the gradient of image intensity, such that diffusion across steep edges in intensity is abolished. The linear problem requires solving a large linear system of equations and several methods have been proposed to significantly speed up computations (Du *et al.*, 2010). Because in the future we want to account for the possibility to include additional non-linear reaction terms, for example to account for the interaction between different colour channels, we adopted an even simpler approach, using an explicit finite differences scheme. A first order Euler integration has been implemented; higher order methods, for example the Runge-Kutta fourth order method, or adaptive time stepping schemes could be implemented in a straight forward manner if desired.

$$P^{(t+1)} = P^{(t)} + \Delta t \cdot D \cdot \left[\left(\frac{P_B^{(t)} - P^{(t)}}{w_B \overline{w_{TB}}} - \frac{P^{(t)} - P_T^{(t)}}{w_T \overline{w_{TB}}} \right) + \left(\frac{P_L^{(t)} - P^{(t)}}{w_L \overline{w_{LR}}} - \frac{P^{(t)} - P_R^{(t)}}{w_R \overline{w_{LR}}} \right) \right] \tag{1.1}$$

where $P^{(t+1)}$ is the probability of the middle stencil pixel to be foreground at time *t+1* and Δt is the chosen time increment to update values $P^{(t)}$ at time *t*. Subscripts L, R, T and B denote probabilities *P* for the left, right, top and bottom neighbour pixels. The diffusion constant *D* is discussed in more detail later.

Weights w in Eq. (1.1) limit diffusion of probabilities and depend on image intensities *I*. We define them as:

$$w = e^{-\alpha\left(I-\bar{I}\right)^2} \cdot e^{-\beta(\nabla I)^2} \tag{1.2}$$

Therefore, the weight depends on the difference of the pixel intensity *I* to the mean $\bar{I}$ of seeded foreground pixels, and the gradient of the intensity to the neighbouring pixel. Parameter α penalises pixels whose intensities are far away from the mean of other pixels classified as foreground. Parameter β penalises pixels located on edges, where intensity gradients are high. Terms $\left(I-\bar{I}\right)^2$ and $\left(\nabla I\right)^2$ are normalised before applying Eq. (1.2). Note that $1/(2\alpha)$ and $1/(2\beta)$ are the variances of the corresponding Gaussian function which makes their interpretation easier. Terms $\overline{w_{TB}}$ and $\overline{w_{LR}}$ in Eq. (1.1) are averages of respective top-bottom and left-right weights *w* (Eq. (1.2)). These terms arise from approximating second derivatives in the diffusion equation.

Stability of solution

In order for our scheme to be numerically stable we demand that

$$\frac{D \cdot \Delta t}{\Delta x^2} \square \frac{1}{4} \tag{1.3}$$

We set D to be one tenth of the limit for stability which in terms of segmentation provides sufficiently good results and is fast.

$$D \cdot \Delta t = \frac{1}{40} \cdot \min \left\{ w_B \overline{w}_{TB}, w_T \overline{w}_{TB}, w_L \overline{w}_{LR}, w_R \overline{w}_{LR} \right\} \tag{1.4}$$

The convergence criterion for Eq. (1.1) utilizes the relative error computed between two successive iterations t-1 and t (Eq. (1.5)) for foreground probabilities.

$$r_{FG}^{(t)} = 2 \left| \frac{P_{FG}^{(t-1)} - P_{FG}^{(t)}}{P_{FG}^{(t-1)} + P_{FG}^{(t)}} \right| \tag{1.5}$$

P_{FG} and P_{BG} are solutions of (1.1) for foreground and background seeds respectively. The iterative solver is stopped when relative error r_{FG} drops below desired level.

Computing probabilities and getting final segmentation

We could set an arbitrary threshold level for the probability of being foreground, but a much better approach is to solve (1.1) for probabilities of being foreground and the probability of being background separately and then compare them. This can be easily obtained by swapping the initial pixel classification and solving the problem again. The final segmentation result can be obtained from (1.6)

$$I_{seg} = \begin{cases} 1 & P_{FG} \geq P_{BG} \\ 0 & P_{FG} < P_{BG} \end{cases} \tag{1.6}$$

I_{seg} is segmented binary image.

This approach can be easily extended to segment multiple touching objects, where probability maps P_{FG} are computed for each object and then compared to each other. The largest probability defines the output class for the pixel. Fig. 1 shows a “neutral” segmentation of multiple foreground objects where we assumed that the input image is homogeneous (uniformly white). Exactly one pixel in the middle of each edge of the image is assigned to a different class (probability of affiliation to class c explicitly set to 1, P_{FGC} = 1). Further results (after applying Eq. (1.6)) for differently located initial seeds are shown in Fig. 1.

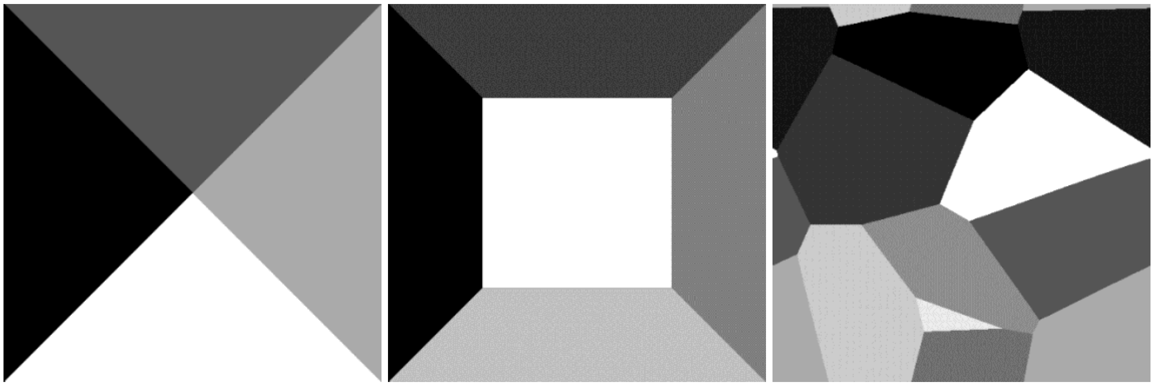

Fig. 1. Results of segmentation of uniformly white image with single pixels assigned to different classes. First image (from left): four single pixel seeds located at the middle of each edge of the image. Second image: five single pixel seeds, four located at the middle of each edge, fifth in the centre. Third image: ten randomly distributed single pixel seeds.

Algorithm improvements

We propose several adaptations to the Random Walk algorithm for our specific application.

Local mean feature

Using an average of intensity of labelled pixels (Eq. (1.2)) can lead to improper segmentation, especially if there are local peaks of intensity within the object. To overcome this problem we propose smoothing the weights domain (1.2) by replacing the global mean $\bar{I}$ with its local equivalent $\bar{I}_{loc}$:

$$\bar{I}_{loc}(x,y)=\frac{\sum_{j=-N/2}^{N/2}\sum_{i=-N/2}^{N/2}F(i,j)I(x-i,y-j)M(x-i,y-j)}{\sum_{j=-N/2}^{N/2}\sum_{i=-N/2}^{N/2}F(i,j)M(x-i,y-j)} \qquad (1.7)$$

F(*i,j*) is constant square convolution kernel, *N* pixels wide and *F* = 1 for $i,j\in[-N/2,N/2]$. *M* is the binary image (of size of *I, M=1* for masked area) that denotes our area of interest, which is usually the object being segmented. We consider M to be a rough segmentation of the image covering the whole object, which is typically obtained when the active contour algorithm is used as preliminary segmentation. It is recommended that the mask is larger than the object to obtain a proper segmentation of its edges. Fundamentals of this process are depicted in Fig. 2.

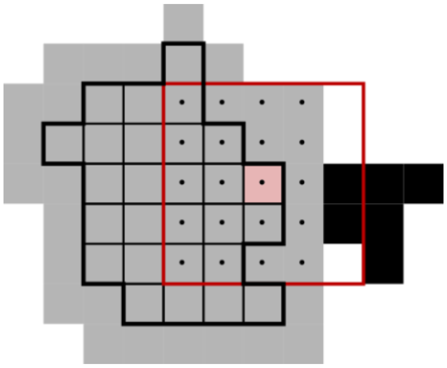

Fig. 2. Explanation of local mean feature. Black open squares belong to the object to be segmented. Black filled squares indicate a nearby other object. The grey area is a binary mask obtained by an active contour segmentation of the object of interest. Red square represents a convolution kernel with a width of five pixels. The local mean (1.7) *computed for the pixel shaded in red, which is at the centre of the convolution kernel, is obtained by considering the 20 pixels which result from the intersection of the convolution kernel and the binary mask. These pixels have been marked by dots.* Note that the nearby object (filled black squares) does not contribute to computing the mean.

Equation (1.1) is solved independently for foreground and background probabilities. Computing the local mean as outlined in Fig. 2 is only applied to the foreground probability. For background probabilities the global mean is used:

$$w^{(FG)} = e^{-\alpha\left(I-\bar{I}_{loc}\right)^2} \cdot e^{-\beta(\nabla I)^2} \tag{1.8}$$

$$w^{(BG)} = e^{-\alpha\left(I-\bar{I}\right)^2} \cdot e^{-\beta(\nabla I)^2} \tag{1.9}$$

Both means are computed only for labelled pixels, for foreground they are defined by binary mask *M*, for background they can be freely scribbled or obtained by inversion of an enlarged *M*.

Auto generating seed pixels

The mask *M* is also used for generating seed pixels for background and foreground. Because we assumed that it covers the whole object without catching small details on its edges like cavities or protrusions, it should first be shrunk to label only pixels that belong to the object and then be expanded and inverted to label background. The ratio of shrinking/expanding should be chosen to avoid producing false labels, e.g. when a contracted mask still covers some of background pixels or vice versa. For complex shapes, simple proportional scaling by e.g. morphological erosion leads to erasing parts of the mask, as depicted in Fig. 3. To overcome this problem, we reused the ECMM (Tyson *et al.*, 2010) method utilised in the ANA module to shrink/expand binary masks. This approach guarantees that protrusions like that one shown in Fig. 3 will still be seeded, even if the distance by which the contour is contracted is comparable to the length of protrusions. The power of shrinking can be made dependent on local curvature and direction of normals that acts against false labelling if cells change their shape rapidly. Some examples of this feature are given in Fig. 4.

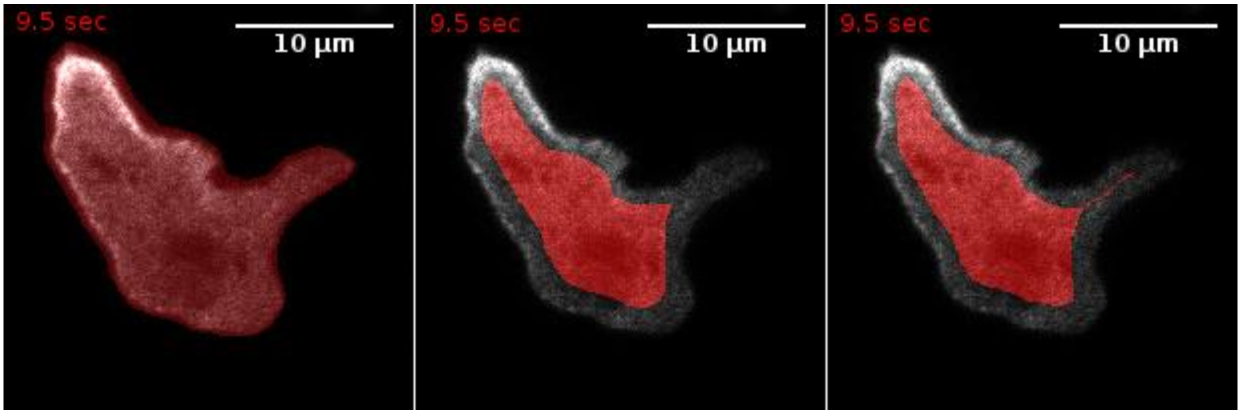

Fig. 3. Examples of mask shrinking by different algorithms to obtain foreground seed pixels. First image shows original mask M obtained from active contour segmentation. Second image shows mask M processed by erosion with 3x3 kernel iterated twelve times. Third image is the same mask but contracted by ECMM method with distance of 15 pixels. Here, seed pixels are retained in the protrusion to the right. Sample from supplementary materials C (SI-C).

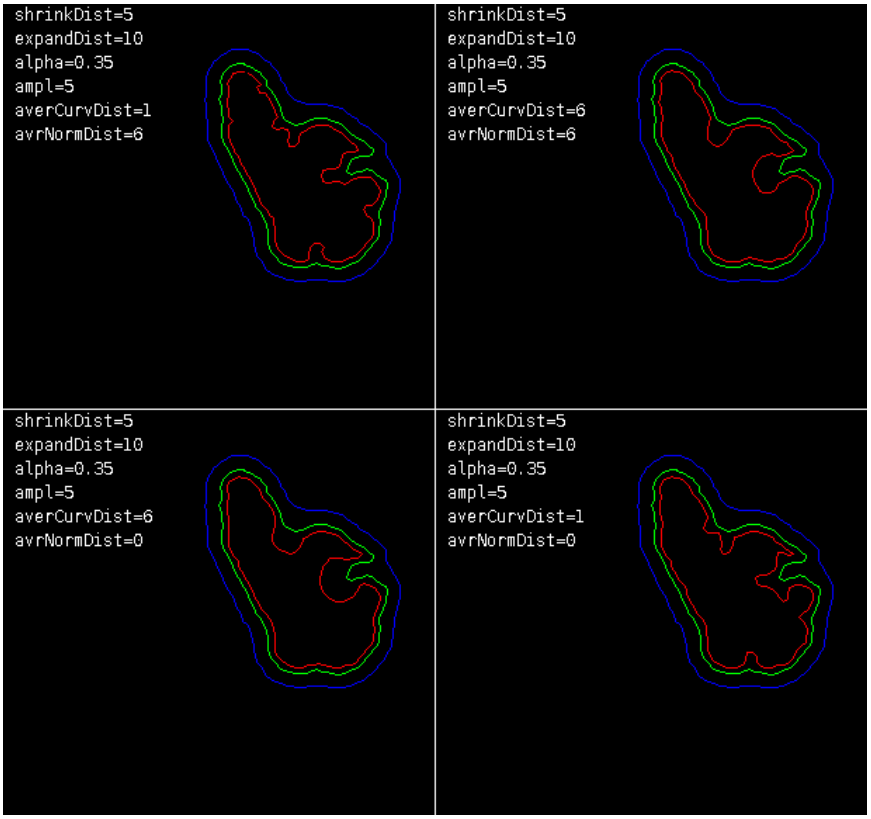

Fig. 4. Contour aware shrinking examples. Original cell contour is drawn in green, red is the original contour constricted with parameters specified on left (related to Random Walk module available from QuimP Toolbar) and blue is the original contour but expanded.

Two pass segmentation

We use two-pass segmentation for getting better results. The first pass computes foreground and background probability maps, which then are utilised in a second pass as input seeds. The second pass returns the final segmented image (Eq. (1.6)). Both passes use exactly the same algorithm described in chapters above. The improvement results from starting with an increased number of

foreground/background seeds when performing the second pass. As the remaining area between the foreground and background seeds, which is to be segmented, is smaller, a steeper gradient in P is obtained. This helps to increase the contrast at the boundary of foreground and background, and with it the sensitivity of the method.

Seeds for second pass are computed in the following way:

$$I_{FG}^{(2)} = \begin{cases} 1 & P_{FG}^{(1)} > 10^{20} \cdot P_{BG}^{(1)} \\ 0 & \text{otherwise} \end{cases}$$
$$I_{BG}^{(2)} = \begin{cases} 1 & P_{BG}^{(1)} > 10^{20} \cdot P_{FG}^{(1)} \\ 0 & \text{otherwise} \end{cases} \quad (1.10)$$

where $P_{FG}^{(2)}, P_{BG}^{(2)}$ are seed pixels for foreground and background for second seed respectively.

References

Du,C.J. *et al.* (2010) Interactive segmentation of clustered cells via geodesic commute distance and constrained density weighted Nyström method. *Cytometry,* **77A**(12), 1137–1147.

Grady,L. (2006) Random walks for image segmentation. *IEEE T Pattern Anal,* **28**(11), 1768–1783.

Tyson,R.A. *et al.* (2010) High Resolution Tracking of Cell Membrane Dynamics in Moving Cells: an Electrifying Approach. *Math Model Nat Pheno,* **5**(1), 34-55.
